# Supplementary material for: Potential enablers for the implementation of multiple family group therapy intervention in the lower Manya Krobo District, Ghana: Perspectives of multiple stakeholders
Source: PLOS Glob Public Health. 2026 Jan 16;6(1):e0005620. doi: 10.1371/journal.pgph.0005620 (PMC12810799; doi:10.1371/journal.pgph.0005620)
Supplement: S1 Text — (DOCX) [file pgph.0005620.s001.docx]

**Guide for FGD on the enablers and barriers to implementing a Multiple Family Group Therapy in Ghana: Health care professionals**

**Introduction**

Thank you for agreeing to participate in this interview. My name is [Interviewer Name], and I am a researcher from [Institution]. Today, we will be discussing your perspectives on implementing Multiple Family Group Therapy (MFGT) for adolescents living with HIV/AIDS here at Atua Government Hospital. This conversation will help us understand potential challenges and opportunities for this program.

The interview will take approximately 45-60 minutes. I'll be asking you questions about your thoughts on this type of therapy, possible barriers to implementation, and suggestions for making it successful. There are no right or wrong answers – we are interested in your honest opinions and experiences.

With your permission, I will audio-record this conversation to ensure I don't miss any important information. All your responses will be kept confidential, and your identity will not be revealed in any reports resulting from this study. You can skip any questions you don't want to answer, and you can stop the interview at any time.

Do you have any questions before we begin?

**Demographic Information**

1. What is your age?
2. What is your gender?
3. What is your professional role and specialty? (e.g. nurse, counselor)
4. How many years of experience do you have in working with adolescents living with HIV and their families?
5. What is the highest level of education you have completed?
6. What is the approximate size of the patient population you serve, including ALHIV?
7. Do you have any additional training or specialization in the area of mental health and psychosocial support?
8. Could you tell me a bit about your role here at Atua Government Hospital?
9. How long have you been working with adolescents living with HIV/AIDS?
10. Have you had any experience with group therapy or family-based interventions before?

**Interview Questions**

**Intervention Characteristics**

1. What are your initial thoughts about Multiple Family Group Therapy as an approach for addressing mental health issues among adolescents living with HIV/AIDS?
2. What aspects of this therapy do you think would work well in this setting? What aspects might be challenging?
3. How do you think MFGT would need to be adapted to be most effective for adolescents and families in this community?
4. What would make this type of therapy appealing or unappealing to adolescents and their caregivers?

**Outer Setting**

1. What community factors might hinder the implementation of MFGT for adolescents living with HIV/AIDS?
2. How do you think stigma in the community might affect participation in a group therapy program?
3. What transportation or access challenges might families face in attending regular therapy sessions?
4. How do cultural beliefs or practices in this community influence how mental health support is viewed or accepted?

**Inner Setting**

1. What resources or supports currently exist at this facility that would help implement MFGT?
2. What resource constraints or limitations might make it difficult to implement this program?
3. How would you describe the readiness of this facility to implement a new mental health intervention?
4. How might this program fit within or compete with other priorities at this facility?

**Characteristics of Individuals**

1. What concerns might adolescents have about participating in group therapy with their caregivers?
2. What concerns might caregivers have about participating?
3. How might disclosure status affect which adolescents could participate in this program?
4. What skills or training would healthcare providers need to successfully facilitate MFGT sessions?

**Concluding Questions**

1. What do you think would be the biggest challenge in implementing MFGT here?
2. What would be the biggest opportunity or benefit?
3. Is there anything else you think we should consider regarding the implementation of MFGT for adolescents living with HIV/AIDS at this facility?

Thank you very much for your time and insights. Your perspectives will be valuable in helping us understand how to effectively implement mental health support for adolescents living with HIV/AIDS.
