# Supplementary material for: Potential enablers for the implementation of multiple family group therapy intervention in the lower Manya Krobo District, Ghana: Perspectives of multiple stakeholders
Source: PLOS Glob Public Health. 2026 Jan 16;6(1):e0005620. doi: 10.1371/journal.pgph.0005620 (PMC12810799; doi:10.1371/journal.pgph.0005620)
Supplement: S3 Text — (DOCX) [file pgph.0005620.s003.docx]

**Guide for Focus Group Discussion/Interviews on the Enablers and barriers for Multiple Family Group Therapy Implementation Study in Ghana: Adolescents living with HIV.**

**Introduction**

Welcome and thank you all for joining us today. My name is [Facilitator Name], and I'll be leading our discussion today. This is [Assistant Name], who will be helping me and taking notes.

We have invited you here because your perspectives are very important for our research. We are exploring the possibility of implementing Multiple Family Group Therapy (MFGT) at Atua Government Hospital to support the mental health of adolescents living with HIV/AIDS and their families.

During this discussion, we want to hear your thoughts about the potential benefits and challenges of such a program. There are no right or wrong answers, and we value all of your opinions. We encourage everyone to participate and respect each other's views, even if they differ from your own.

This discussion will take about 60-90 minutes. With your permission, we will audio-record this conversation to ensure we capture all your valuable insights. Everything you share will be kept confidential, and your names will not be used in any reports.

Before we begin, let's go around and introduce ourselves**. [Conduct brief introductions for background information]**

**Ground Rules**

- Everyone's views are important and valid
- Please speak one at a time so we can hear everyone clearly
- Respect the confidentiality of what is shared in this room
- Participation is voluntary – you can choose not to answer any question
- There are no right or wrong answers – we want your honest opinions

Does anyone have any questions before we begin?

**Discussion Questions**

**Opening Questions**

1. What are your experiences with health programs or support groups at this facility?
2. What kinds of challenges do [adolescents living with HIV/AIDS face in this community

**Intervention Characteristics**

1. We'd like to tell you a bit about Multiple Family Group Therapy. [Brief explanation of MFGT]. What are your initial thoughts about this type of program?
2. What would make this type of program attractive or helpful to you?
3. What aspects of this program might be difficult or concerning for you?
4. If you were to participate in such a program, what topics or skills would you want it to cover?

**Transportation and Logistics**

1. What challenges might you face in attending regular meetings at the hospital?
2. What would make it easier for you to attend these meetings regularly?
3. When would be the best time and day for such meetings? How long should they be?
4. What type of space or environment would make you feel comfortable participating in group discussions?

**Stigma and Confidentiality**

1. What concerns might you have about privacy or confidentiality in a group program?
2. How might participating in this program affect how others in the community view you?
3. What could be done to address concerns about privacy and stigma?
4. How would you feel about meeting in a group with other families affected by HIV?

**Group Dynamics**

1. How would you feel about sharing experiences and discussing challenges in a group setting?
2. How would you feel about participating in discussions with your caregiver present?
3. What would help you feel comfortable speaking up in a group setting?

**Program Implementation**

1. What might prevent people from joining or staying in this program?
2. What support would you need to participate regularly in this program?
3. How should families be invited to join this program?
4. What would make this program successful in this context?
5. What interactive activities would you want the team to incorporate in the program?
6. What competing interest may disrupt your continued participation in the program?

**Closing Questions**

1. What do you see as the biggest challenge to implementing this program?
2. Is there anything else you would like to share about implementing this type of program?
