# Supplementary material for: Potential enablers for the implementation of multiple family group therapy intervention in the lower Manya Krobo District, Ghana: Perspectives of multiple stakeholders
Source: PLOS Glob Public Health. 2026 Jan 16;6(1):e0005620. doi: 10.1371/journal.pgph.0005620 (PMC12810799; doi:10.1371/journal.pgph.0005620)
Supplement: S1 Data — (DOCX) [file pgph.0005620.s005.docx]

**Focus Group Discussion Transcript**

**Healthcare Workers**

PARTICIPANT DEMOGRAPHICS

[Interviewer]

Before we begin the main questions, I'd like to collect your biographical data. For confidentiality purposes, I won't use your names. Instead, I'll label you as Respondent 1, 2, 3, 4, and 5. Let's start with Respondent 1. What is your age?

[R1]

I'm 32 years old.

[Interviewer]

Your gender?

[R1]

Male.

[Interviewer]

What is your professional specialty? What do you do here?

[R1]

I'm a nurse.

[Interviewer]

How many years of experience do you have working with adolescents living with HIV in this Atua HIV facility?

[R1]

Four to five years.

[Interviewer]

What is your highest level of education?

[R1]

Diploma.

[Interviewer]

What type of healthcare facility is this in Atua?

[R1]

It's a district health facility, not municipal.

[Interviewer]

Have you received any additional training in mental health?

[R1]

No.

[Interviewer]

What about training in psychosocial support or counseling?

[R1]

Yes.

[Interviewer]

Was it through in-service training or continuous professional development?

[R1]

Both—in-service training and continuous professional development (CPD).

[Interviewer]

Thank you. Now, Respondent 2, what is your age?

[R2]

40.

[Interviewer]

Your gender?

[R2]

Female.

[Interviewer]

What's your professional role?

[R2]

I'm a nurse.

[Interviewer]

How many years have you worked in this facility, specifically with adolescents living with HIV?

[R2]

I'm new to this unit. This is my second week here.

[Interviewer]

What is your highest level of education?

[R2]

Degree.

[Interviewer]

Have you received any training in mental health?

[R2]

Yes, we had it here through a workshop.

[Interviewer]

How many times have you attended such training?

[R2]

Once.

[Interviewer]

Have you received additional training in psychosocial counseling or psychosocial support?

[R2]

No.

[Interviewer]

Thank you. Respondent 3, what's your age?

[R3]

I'm 30.

[Interviewer]

Your gender?

[R3]

Male.

[Interviewer]

Your professional role?

[R3]

I'm a nurse and a prescriber.

[Interviewer]

How many years have you worked in this HIV unit, especially with adolescents living with HIV?

[R3]

I've worked in this unit for two years, but I've been at the facility for four to five years.

[Interviewer]

Have you received additional training in mental health?

[R3]

Yes, through in-service training. I've had about three training experiences.

[Interviewer]

Have you received training in psychosocial counseling or psychosocial support?

[R3]

Yes, through professional academics and CPD. During my nursing training, I had mental health modules and even went to Pantang for practical training.

[Interviewer]

So you have extensive knowledge in mental health?

[R3]

Yes, please.

[Interviewer]

Respondent 1, you also had similar training during your nursing education, correct?

[R1]

Yes, we even went for clinical affiliation. I didn't mention it earlier because I didn't fully understand the question.

[Interviewer]

Respondent 4, what's your age?

[R4]

59 years.

[Interviewer]

Gender?

[R4]

Female.

[Interviewer]

What is your role here?

[R4]

I am a general nurse working in the counseling unit, so I serve as a counselor.

[Interviewer]

How many years have you been working with adolescents living with HIV?

[R4]

I've been working here for nine years, all of it engaging with adolescents living with HIV.

[Interviewer]

Have you received training in mental health?

[R4]

Yes, during my nursing training at Ankaful. I know much about mental health.

[Interviewer]

Have you attended any workshops on mental health since your training?

[R4]

No, I haven't attended workshops specifically on mental health, though I've attended workshops on other topics.

[Interviewer]

Respondent 5, what's your age?

[R5]

I'm 50.

[Interviewer]

Your gender?

[R5]

I'm a woman.

[Interviewer]

What role do you play here?

[R5]

I'm a mentor mother.

[Interviewer]

What is your highest level of education?

[R5]

Junior Secondary School (JSS).

[Interviewer]

How many years have you worked here helping children with HIV and their parents?

[R5]

Sixteen years.

[Interviewer]

Have you received training in mental health?

[R5]

Yes, we went for a workshop in Koforidua once.

[Interviewer]

Have you received training in counseling and psychosocial support?

[R5]

Yes, please.

# MAIN INTERVIEW QUESTIONS

[Interviewer]

What interventions or initiatives currently exist to improve the mental health of adolescents living with HIV and their caregivers in this facility or community?

[R3]

During our management of patients, we realized that adolescents attending clinic alongside adults often encounter these same adults in the community. This caused them to feel shy and reluctant to discuss their concerns openly. To address this, the facility decided to set aside specific days for adolescents. We have Wednesdays dedicated exclusively to adolescents. This helps prevent psychological and emotional problems by creating a more comfortable environment where they can freely express themselves.

[R1]

We also engage them every morning with health education. We provide psychological support regarding their condition and medications. Every morning when they report to the clinic, all unit staff educate them on what they need to know. We give them opportunities to ask questions about anything bothering them at home—nutrition, medications, or if someone is abusing them.

[R4]

One observation I've made is that the atmosphere here is very friendly and cordial. When adolescents come in and you're too harsh, some might decide not to return. But here, from the staff to everyone else, everybody is friendly. This eases their tension, and they feel free to come and complain about anything bothering them.

[R5]

Sometimes we create support groups for them so they can know each other and support one another. We have the Adolescent Differentiated Service Delivery (DSD) support group.

[Interviewer]

When adolescents come, do you have tools to assess their mental health? How do you identify specific mental health problems like depression, anxiety, or suicidal tendencies? Is there any standardized way of assessing their mental status before providing counseling?

[R4]

When they come, we give them appointment cards. If someone defaults, we probe to find out why. Sometimes they reveal their problems, and we help them solve these issues. Sometimes they lack food or money, which prevents them from taking their drugs. We document their needs in case an NGO wants to help.

[R3]

We rely mainly on clinical assessment. Through our ongoing engagements, we can tell how alert and oriented they are. Sometimes we notice they're falling back or not keeping up, indicating a problem somewhere. Often, it's related to family relationships. We trace these issues to their root causes and address them as much as possible. Once the underlying problems are resolved, they return to themselves and become more engaged.

[R1]

There is no specific tool we use for assessing their mental health. Our most reliable tool is history taking. As we engage them, we take their domestic histories to understand how they're faring at home. We also provide privacy so they can comfortably share whatever is bothering them with the counselor.

[Interviewer]

Have any adolescents or caregivers directly reported experiencing depression, excessive worry, or suicidal ideation? Have you had instances where they've reported emotional challenges since the HIV diagnosis?

[R1]

Personally, I haven't had such complaints. The main complaint I receive is drug fatigue, which is common among all our patients. They complain about being tired of taking medications. We use our counseling skills to encourage adherence, focus on nutrition, and engage their caregivers to support them. However, I haven't had complaints about suicidal ideation.

[Interviewer]

Respondent 5, you mentioned support groups. Do these meetings address mental health problems? Are caregivers allowed to attend? Do you think these meetings effectively address mental health?

[R5]

Sometimes we ask them to come with their parents or caregivers. Sometimes they come alone. When they come, we provide education on mental health and nutrition. We have different topics we cover each time.

[Interviewer]

What specific education do you provide related to mental health?

[R1]

We discuss sexual exploitation and sexual abuse, especially for those at vulnerable ages who might be targeted by predators. We educate them to always be on guard and report to their parents, caregivers, or us. We've made our telephone numbers available for reporting any inappropriate touching. For those complaining about drug fatigue, we conduct home visits using our mentor mothers, models of hope, and staff. We visit to monitor the situation because some don't have food before taking medications. We involve their caregivers to make the environment more conducive for medication adherence.

[Interviewer]

Respondent 3, you mentioned that some adolescent problems stem from family issues. Do you think an intervention focusing on the family would be helpful?

[R3]

Yes, I absolutely agree that a family-focused intervention would be helpful. Many issues bothering these adolescents come directly from family—financial issues, spirituality, religion, and so on. The more attention we pay to these families and the more we engage with them, the better. We've had experiences inviting adolescents with their caregivers for sessions, and the discussions went very well. I believe an intervention that focuses directly on caregivers or the family would effectively support them.

**Introduction of Multiple Family Group Therapy (MFGT)**

[Interviewer]

Based on your responses, I want to introduce the intervention we're here to implement in Lower Manya Krobo: Multiple Family Group Therapy (MFGT). This intervention brings different families together—specifically focusing on adolescents and their direct caregivers who are aware of the diagnosis. We want to avoid stigma, so we won't involve extended family members who might not know about the diagnosis.

We'll bring these families together to discuss: family relationships, respectful communication, problem-solving and conflict resolution, how adolescents' voices can be heard in the family, stress management, social support within families, and roles and responsibilities.

Yesterday, I spoke with some adolescents. One 16-year-old shared that when her mother scolds her in public, she feels very embarrassed. This shows that while the child has built emotional resilience regarding HIV, the relationship with her mother fuels her depression.

We're going to train some healthcare workers interested in leading these sessions. The intervention will have 14 sessions over 14 weeks, meeting once weekly on Sundays after church for two hours. We'll cover topics ensuring that beyond HIV-related stress, children don't suffer additional trauma from family interactions.

[Interviewer]

What practical activities should be incorporated to make the training more interesting, engaging, and interactive?

[R2]

I agree with Respondent 4 about vocational training—teaching them soap-making, how to prepare sobolo and other drinks. Simple things they can do in the community. This engagement would be beneficial.

[R3]

I'd also emphasize personal hygiene. Girls form the majority of our population. Some don't have sanitary pads, perfumes, or even soap for bathing. Focusing on hygiene would be an excellent intervention.

[Interviewer]

What existing healthcare policies would support this intervention? Are there HIV care policies that could support our family-focused mental health intervention?

[R3]

There's the Community Refill Program. Clients who are doing well with suppressed viral loads and no underlying conditions can receive medications in the community through specific clinics and health centers. The program creates groups of ten who meet monthly for discussions on topics like partner disclosure, viral load, adherence, and treatment support. Some families or caregivers invited to our intervention may already know about this program. Since they're familiar with such group-based support, adding a similar intervention focusing on themselves and their caregivers would be sustainable. We're building on existing foundations.

[Interviewer]

Can this MFGT program be integrated into existing HIV care so it continues after we leave?

[R1]

Yes, there's high possibility. Once you train us as staff, we'll have the knowledge. Even after the program officially ends, we can continue the intervention.

[Interviewer]

Besides staff training, what else can ensure sustainability?

[R1]

We'll incorporate it into our regular routines. As staff transition, we'll train new staff members, ensuring the knowledge transcends to future healthcare workers.

[R4]

We also need NGO support because it requires money. When people come, we need to provide transport allowances. For sustainability, there should be ongoing funding so we can provide refreshments and support to clients and adolescents.

**[Interviewer]**

What resources can be leveraged now to support implementation? After we leave, what available resources can ensure sustainability?

[R3]

Currently, I can speak to human resources. We're available and always ready to help keep it going.

[R2]

The physical structures here are inadequate. This space is too small. If we start getting 50 to 100 adolescents and caregivers, this place can't accommodate them. If we could have expansion and make the unit more comfortable with air conditioning, bottled water, and adequate space, people would be comfortable and even invite others. When the place is hot and uncomfortable, nobody will return.

[Interviewer]

What skills and training would you need to successfully facilitate this intervention?

[R1]

Once you organize a workshop for us, we're readily available to attend.

[Interviewer]

How many training sessions would adequately equip you? Would meeting once be enough?

[R4]

We need learning materials—things we can see to understand better. Sometimes rushing through content prevents understanding. Maybe two or three days of training would be okay with materials we can reference.

[R1]

Audio-visual learning materials on pen drives would help. We could play them on televisions for review and revision.

[Interviewer]

How can we ensure your commitment to this program? How can we secure your loyalty until the program is successfully implemented?

[R3]

Healthcare workers as facilitators could send final reports to the team. We could seek client consent for photographs as proof. Attendance forms could be used where families and healthcare providers endorse participation—all available to prove we're supporting the program.

[R1]

Personally, I believe in motivation. We have routine work already; this would be additional responsibility. If we could be motivated with a small token at the end of each week or month, it would boost morale. We're ready to deliver on this mandate with a little something to encourage us.

[R2]

Besides monetary motivation, improving our work environment would boost morale. We're bringing adolescents and caregivers on Sundays. Without motivating staff, we'll all be reluctant. I live in Akosombo; others live in Somenya, Agomenya, Nuaso—quite a distance. If we see structural improvements in our unit, our morale would increase. We'd come whenever called.

**[Interviewer]**

We've secured a location we're planning to renovate for holding sessions. Respondent 1, could you explain this to the group?

[R1]

Professor Adjorlolo mentioned they'd like to renovate a temporary unit for the program. Whenever they visit, they'll use that room for engaging members. After they leave, the hospital will benefit from the renovated office. We've identified two places: one at the female ward and one behind the conference room. Initially, they'll work on the one at the female ward as their office.

[R1]

I believe scheduling solves this. We already have an existing timetable; we just need to reschedule. Through planning, everyone will know their responsibilities and timing for engaging adolescents. This shouldn't be a problem.

[Interviewer]

What cultural values in Lower Manya Krobo can support our intervention? Are there cultural practices that support bringing different families together?

[R1]

Building on the festival idea—each year during festivals, planners invite healthcare workers for HIV screening. Beyond screening, we could involve stakeholders from the traditional council, district health directorate representatives, and youth group leaders. We could engage these stakeholders more comprehensively. The festivals are in October-November every year.

[Interviewer]

What challenges might you face as healthcare providers during program implementation?

[R4]

Stigma, discrimination, and disclosure are issues. The Krobo area is known as an HIV area. I'm concerned about the program's name so people won't identify participants as 'HIV people meeting.' It's a very sensitive area.

[R1]

My fear is people not showing up. Sometimes they promise to come, but on the day, they cite various challenges. We can't force people to attend.

[Interviewer]

What can we do to motivate them and ensure they keep their commitments?

[R1]

First, we need to verify their telephone numbers and contact details. Second, adolescents and relatives must be assured their transport costs will be covered. Third, when they come, refreshments should be provided. Fourth, we should respect the time frame—if it's two hours, stick to two hours.

[R2]

I anticipate that family members will start bringing financial burdens to healthcare workers. Parents and relatives will realize participants receive transport allowances and food, so they'll start requesting help with school fees and other issues.

[Interviewer]

What can prevent this from happening, especially after we leave?

[R2]

Before starting, on the first day, we should clearly explain that this project runs for a specific period. After that, the implementing organization leaves. This isn't a lifetime program to take care of entire families. We should repeat this message at every meeting and inform caregivers so they don't develop expectations about school fees and other financial support.

[Interviewer]

How can we overcome community stigma? Won't people question why they're coming to the hospital every Sunday?

[R3]

It depends on the education we provide. If our education is in-depth and our engagement friendly and cordial, I believe individuals can overcome internalized stigma. Once they overcome that, they won't focus on what others say. By continuously bridging the gap and letting them know the benefits of attending with their caregivers, we'll reduce the fear of stigma.

[Interviewer]

When I spoke with caregivers, several raised confidentiality concerns. Since healthcare workers will lead the program and families will share experiences, they want assurance that their information will remain confidential. Do you think confidentiality will be challenging?

[R4]

For healthcare workers, I don't think it's a problem. However, other people around the hospital—those on admission and other staff—might overhear things.

[R2]

All healthcare workers went through training where confidentiality was a core component. Personally, I don't think any healthcare worker would breach confidentiality. For healthcare workers, you're assured.

[R1]

Before this intervention, we've been dealing with these adolescents without any confidentiality breaches. This project won't change anything. Our work is independent of the project. The professionalism we maintain will continue.

[Interviewer]

How can we organize sessions so discussions stay within the room and aren't overheard?

[R1]

The room is completely enclosed. It has windows opening outside, not toward the ward. I strongly doubt any discussion inside will be heard outside. Confidentiality is assured—there's no way issues will leak from that space.

[Interviewer]

Do you anticipate any organizational challenges within Atua Government Hospital that could affect implementation?

[R2]

I'm anticipating that in the future, they might reclaim that space for another ward or project. This has happened before.

[R1]

That's a valid concern, but we have endorsement from the administrator and medical superintendent—the main governing bodies. Once they've released the room, at least until the project ends, they won't reclaim it.

[R3]

I think we should reconsider the venue. The conference room is better than the ward option. At the conference room, people are less likely to perceive participants as HIV-related. It's more like an administrative route.

[R4]

There are also multiple routes to that area—you can pass through different directions. No one will necessarily see you entering.

[Interviewer]

When I spoke with caregivers, they suggested that family-related topics should be facilitated by trained caregivers, while health-related topics should be facilitated by healthcare workers. Do you agree?

[R3]

I disagree. However, if we allow them, it should be under supervision. As trained healthcare workers, one challenge we've dealt with is disbeliefs and myths. As healthcare workers providing awareness and better understanding, we can make progress. Without supervision, we may not know what's being discussed.

[R2]

If possible, train both groups—train healthcare workers and also train caregivers to facilitate some sessions under our supervision.

[R4]

There should be variation so it doesn't become monotonous. Allow caregivers to participate in some sessions while we monitor thFinal Comments and Concerns

[Interviewer]

As we conclude, is there anything you'd like to share that hasn't been asked or mentioned?

[R4]

My clients always have problems with food. Taking their drugs is not easy. They need to eat before taking medications. If you can help us with food so they can take their drugs properly, they'll stop defaulting. Their viral loads are always high because they're not taking drugs as prescribed.

[R1]

One challenge I've identified—you're requesting chemistries for all our clients to assess liver functions, kidney functions, cholesterol, and so on. Some, especially adolescents, can't afford these labs. If this project could help cover their chemistries for at least a year, we'd greatly appreciate it.

[R2]

I want to stress vocational training for young people. When we train them to earn money for themselves, they can buy things independently. After the program, they won't depend on others. Vocational training is very important. Also, please consider our structure and push for expansion because this place is too small.

[R3]

Once we commence, the project should clearly spell out to adolescents and caregivers what support is available. If it's vocational training within the 14 weeks, they should know this is part of implementation. We need to educate them about their expectations. If everything is made known and within 14 weeks we all understand our direction, once we end, we'll know they've benefited, healthcare workers have gained knowledge, and we move forward as a group.

[R5]

Also support them with vocational training so they'll have something to do. When they come for their drugs, they might need ten cedis for transportation. We should support them with job training so they can earn money.

[Interviewer]

Thank you all so much. Your comments, views, and ideas have been well noted. Thank you.

END OF TRANSCRIPT
