# Supplementary material for: Potential enablers for the implementation of multiple family group therapy intervention in the lower Manya Krobo District, Ghana: Perspectives of multiple stakeholders
Source: PLOS Glob Public Health. 2026 Jan 16;6(1):e0005620. doi: 10.1371/journal.pgph.0005620 (PMC12810799; doi:10.1371/journal.pgph.0005620)
Supplement: S2 Data — (DOCX) [file pgph.0005620.s006.docx]

**Focus Group Discussion Transcript**

**Caregivers of ALHIV**

INTRODUCTION AND PARTICIPANT DEMOGRAPHICS

[Moderator]

I believe you are all aware of the reason we have met today. The In-charge called you. We know that in most cases, Adolescents living with HIV experience considerable emotional trauma that can lead to depression. Sometimes they enter a state of melancholy and pain when they remember they are carriers or when they think about being on medication for life. This can cause emotional turmoil, which is why we are here today. We want to develop a support system for caregivers of adolescents living with HIV. We need your help because we want to join forces with you to achieve this program. We need your ideas and thoughts so we can implement a successful intervention where you can attend and share your experiences caring for an adolescent living with HIV. We want to know what you go through.

Before we dive into the discussion, I would like to ask each of you some questions to gather information. I'm [redacted] from Accra. We came here to interact with you months ago, so we are back again to continue, just as we promised.

[Moderator]

Can you tell us your age?

[Participant 1]

25 years.

[Moderator]

I can see male features, but for the purpose of this discussion and given current gender issues, I'd like to ask: are you male or female?

[Participant 1]

I'm male.

[Moderator]

What is your level of education?

[Participant 1]

I've completed Junior High School (JHS).

[Moderator]

What is your religious affiliation? Are you Christian, Muslim...?

[Participant 1]

I'm a Christian.

[Moderator]

Are you married?

[Participant 1]

No.

[Moderator]

If you're not married, what is your current status? Have you been married before?

[Participant 1]

No.

[Moderator]

Okay, so you're single. How are you related to the adolescent you brought?

[Participant 1]

She is my little sister.

[Moderator]

How long have you been caring for her? When did you learn that she is a carrier of the virus?

[Participant 1]

She was living with our mother. At the time, I wasn't aware until she came here.

[Moderator]

Since you became aware and started caring for her, how long has it been?

[Participant 1]

About a year.

[Moderator]

Do you live with her alone, or are there other family members?

[Participant 1]

There are other family members.

[Moderator]

In caring for her—her feeding, bringing her for medication—do you receive help from anyone, including family?

[Participant 1]

No.

[Moderator]

The help could be in the form of transportation to the hospital.

[Participant 1]

It's her madam who supports us.

[Moderator]

Which madam?

[Participant 1]

I've forgotten her name.

[Moderator]

Is she at your house?

[Participant 1]

No, she's at her school.

[Moderator]

So her school teacher supports her?

[Participant 1]

Yes.

## Participant 2

[Moderator]

Okay, mama, please can you tell us your age?

[Participant 2]

Sure I can. I'm 70 years old.

[Moderator]

Are you male or female?

[Participant 2]

I'm female.

[Moderator]

What is your level of education?

[Participant 2]

Form 4.

[Moderator]

What is your religious affiliation?

[Participant 2]

I'm a Christian.

[Moderator]

Are you single, married, divorced, or widowed?

[Participant 2]

I'm widowed.

[Moderator]

What is your relationship with the child you came with?

[Participant 2]

She is my grandchild.

[Moderator]

How long have you been caring for the child—feeding, bringing her to the hospital, managing her medication? Since she was confirmed a carrier, how many years have you been caring for her?

[Participant 2]

About 8 years.

[Moderator]

Do you both live alone, or are there other family members living with you?

[Participant 2]

We live in a family house, so there are others.

[Moderator]

Do you get help from anyone in caring for the child?

[Participant 2]

Yes, the child's uncle, her mother's older brother, helps us financially.

## Participant 3

[Moderator]

Auntie, it's your turn now. How old are you?

[Participant 3]

I'm 31 years old.

[Moderator]

Are you male or female?

[Participant 3]

I'm female, but I can say I'm a male too.

[Moderator]

How? Please explain.

[Participant 3]

In the sense that only I take care of the child—no one helps.

[Moderator]

To say that you perform both the duties of a man and a woman. I understand. What is your level of education?

[Participant 3]

I completed JHS.

[Moderator]

What is your religious affiliation?

[Participant 3]

I'm a Christian.

[Moderator]

What is your marital status?

[Participant 3]

I'm single.

[Moderator]

Never married?

[Participant 3]

Yes.

[Moderator]

How are you related to the child?

[Participant 3]

I'm the child's mother.

[Moderator]

Since the child was confirmed a carrier, how long have you been a caregiver?

[Participant 3]

A year and four months.

[Moderator]

Do you both live alone, or are there other family members in the house?

[Participant 3]

We live in a family house, so there are other people.

[Moderator]

Do you get any support from anyone?

[Participant 3]

No.

[Moderator]

When I say support, it's not only financial. Sometimes when you're sad or worried, having someone to encourage and comfort you is also help.

[Participant 3]

I understand, but I don't get any support. I'm the first child amongst my siblings, and they're young, so I don't have anyone to look up to. Rather, they look up to me.

## Participant 4

[Moderator]

Auntie, please, how old are you?

[Participant 4]

40 years.

[Moderator]

Are you male or female?

[Participant 4]

I'm female.

[Moderator]

What is your level of education?

[Participant 4]

JHS.

[Moderator]

What is your religious affiliation?

[Participant 4]

Christianity.

[Moderator]

What is your marital status?

[Participant 4]

I'm widowed.

[Moderator]

How are you related to the child you brought?

[Participant 4]

The child is my child.

[Moderator]

Do you both live alone, or are you also in a family house?

[Participant 4]

We used to live in a family house, but because of my child's situation, I moved out to rent.

[Moderator]

So now you both live alone?

[Participant 4]

No, I live with the child and my two other children.

[Moderator]

Do you get any support from anyone in caring for the child?

[Participant 4]

No.

[Moderator]

When I say help, not just money—anyone who comforts you is also rendering help. Someone might just offer advice.

[Participant 4]

With this virus, should anyone find out, they stigmatize you, so I do not warm up to people.

## Participant 5

[Moderator]

How old are you?

[Participant 5]

I'm 31.

[Moderator]

Are you male or female?

[Participant 5]

I'm female.

[Moderator]

What is your level of education?

[Participant 5]

Senior High School.

[Moderator]

What is your religious affiliation?

[Participant 5]

Christianity.

[Moderator]

What is your relationship with the child?

[Participant 5]

The child is my little sister—she's our last born.

[Moderator]

For how long have you been providing care to the adolescent?

[Participant 5]

About 5 months now.

[Moderator]

Do you receive any kind of support from anyone?

[Participant 5]

Not at all.

*[Moderator provides instructions for discussion format]*

# MAIN DISCUSSION

[Moderator]

As someone who cares for a child living with HIV, does it bother you emotionally? How do you feel? How exactly does it bother you?

[Participant 1]

I don't know how it happened or why the child got infected, and that worries me a lot.

[Moderator]

Do these thoughts make you think a lot?

[Participant 1]

Yes.

[Moderator]

What thoughts do you have? What do you think about?

[Participant 1]

What comes to mind is the thought of finding a drug that will take the virus away completely.

[Moderator]

So you wonder if there is a drug that can cure the child?

[Participant 1]

Yes, I always wonder if there is such a drug, but I don't know if there is or not.

[Moderator]

Do you feel sad, worried, or scared?

[Participant 1]

A lot.

[Moderator]

Tell me some of the things you go through.

[Participant 1]

All my siblings and I have the same mother but different fathers, and this child is the only one with the virus. When I think about that fact, I don't understand, and it worries me.

[Moderator]

So when you think about these things, do you get sad, worried, or do you panic or get scared?

[Participant 1]

Yes. Whenever the child gets sick, the way it happens makes me panic, so I leave the house.

[Moderator]

Do you worry that she might die?

[Participant 1]

Yes, especially when the child gets sick.

[Moderator]

With these experiences you're sharing, has it affected your relationship with the child?

[Participant 1]

I know that God will help us. No, it hasn't changed my relationship with her.

[Participant 2]

For me, when I see the child, I panic and worry because since I learned about the diagnosis and know this fact that it cannot be cured, I worry. I'm already in my 70s, so how can I care for this child until she grows and settles in life? I'm going soon, so when I look at the child, I panic. At times I cry. The child asks why I'm crying, and I just tell her I'm okay.

[Moderator]

How has it affected your relationship with her? Has anything changed?

[Participant 2]

The child asks what the problem is anytime I cry, and I say it's nothing. But I tell her, 'My child, you know our current state, so try and adhere to all you have been told so you would have good standing in life before I leave this world.'

[Moderator]

What are some of your experiences? What are some of the things you go through emotionally, especially looking at the age of your child who carries this virus?

[Participant 3]

I'm pained when I look at my child. When I gave birth, this child never got sick, but the first time of falling sick is when I was informed of the diagnosis. I cry a lot. Even upon seeing the child sleep at night, I cry. At times my child asks why I'm crying, if I don't have money, but I just ask my child to sleep. Honestly, I got sick that day I was told of the diagnosis. It was about a week before I started feeling better.

[Moderator]

Is this worry and panic constant?

[Participant 3]

Anytime I remember my child has this virus. Whenever I see that my child isolates from other children, I try to entertain my child.

[Moderator]

Has the knowledge of the diagnosis increased the love you have for your child?

[Participant 3]

Oh no, the love has always been there, but when I don't see my child, I panic.

[Moderator]

Do you now go an extra mile in caring for your child compared to when there was no virus?

[Participant 3]

There are no changes in the way I care for my child in the sense that I've always gone an extra mile. Even when my child gets ready for school, I have always personally taken my child to school. For the diet, it has changed because with the drugs, if the diet isn't good, my child keeps losing weight. Also now, my child isolates himself because of the weight loss, so I've taken permission from school so my child can sit out for about two weeks just to recover.

[Moderator]

Participant 4, share with us what you also go through emotionally since you were told of the diagnosis.

[Participant 4]

As for what I go through... My child got sick after birth, but the doctors didn't tell me about the virus until recently when the doctors here found out and told me. I was pained because when my child started treatment, that was when I realized it was the same virus that killed my husband. When he was alive, anytime he got sick and visited the hospital, I saw the same drugs amongst the ones he took. It saddens me a lot to know that I already lost my husband to the virus and I'm about to lose my child too. For this reason, my child is the apple of my eye. If you touch my child, you have tampered with my eyes. My child being sick... At times when I see my child, I feel this gut-wrenching feeling because this child is my only female child. Looking at the fact that the virus would just take her away... It worries me.

[Moderator]

You mentioned that you lived in a family house but moved out because you didn't want anyone stressing her. How was the behavior of people in the house towards her? How did they treat her?

[Participant 4]

Even before the doctors found out about her state, she had an outbreak of rashes on her skin. When they're playing, the other parents pull their kids away from her. Because of that, she doesn't mingle with others till now. When she comes back from school, we're always inside our room. Right from the shop, we go into our room because she has also seen how other people treated her, so she doesn't play with other kids.

[Moderator]

Is it the outbreak of rash on her skin that made people avoid her, or is it something else?

[Participant 4]

Well, I think it's the rash on her skin that is making people stop their kids from playing with her.

[Moderator]

Since you were told of her diagnosis, has your relationship with her changed compared to how it was before?

[Participant 4]

Just that her diet and feeding have changed because of the drugs. She can wake up at dawn hungry, so I always have food or drinks in the fridge ready to be warmed whenever she needs to eat.

[Moderator]

Participant 5, what do you have to say concerning the question?

[Participant 5]

To be honest, when I was told, it worried me a bit.

[Moderator]

Did you believe it or not?

[Participant 5]

Oh, I believe it because from the onset, our mother was on the ARVs, and about 13 years ago, I was the only one she told and brought here. It was my stepfather who had the virus first but didn't tell my mom. She found out later, so when I was informed, I wasn't surprised really. I don't really feel anything because my mom has already educated me a lot, and whenever I come with her here, we are educated, so it's like I'm already used to the fact.

[Moderator]

Because your mom has it and now your sister, so it's not new to you?

[Participant 5]

Yes. Secondly, after the diagnosis, her diet changed. I make sure that she has a balanced diet.

[Moderator]

Do you not get sad knowing that your younger sister has this? Don't you worry about whether your sister can grow well?

[Participant 5]

My worry is about her education. She's in JHS 3 now, so I worry about how she can take her drugs when she goes to senior high school. The last time I came for her drugs, the nurse here asked me how I feel about it. My response was: How is she going to be able to take her drugs? Will she get the time or even remember to take her drugs? That is what's important to me. I was told that every school has a nurse, so we can ask the nurse to help us in that regard. That's what worries me. Also, at times she gets quiet and isolates herself. When I see that happen, I try to engage her in activities—go and get water for me, or do this. I don't ask her what is wrong because if you do, that makes her sadder. But if you engage her, like asking her to fold the things in my wardrobe, that will get her mind off things.

[Moderator]

When you see her thinking or getting sad about her status, how does that make you feel as a big sister?

[Participant 5]

That will make you feel bad a little, in the sense that we're all having a good time, then all of a sudden she isolates herself. She has always been like that, even before her diagnosis. Sometimes before, her tongue had some abnormalities and her skin too, so I take her to the pharmacy to get drugs and mouthwash. I make sure she brushes her teeth twice and uses the mouthwash. Because of that, at school she's not sociable already before the diagnosis, so sometimes I feel sad a little, but it's all about encouragement.

[Moderator]

Since you found out about the diagnosis, has it brought any changes in your daily activities? Like the time you report to work or go to the market? For instance, you used to go to the market early, but now you have to wait and give your child drugs before going.

[Participant 2]

For me, it brought changes from the beginning because when we found out, we didn't inform her, so taking the drugs on time and regularly was my responsibility. It affected my daily activities, but since we told the child and she's aware, she's able to adhere to the time, so I've gotten a bit of rest and freedom.

[Participant 3]

My own is that I give my child the drugs at night before bed. It has not brought any changes to my daily activities. For my child's preparation for school, I wake up early to do that.

[Participant 4]

For me, it hasn't brought any drastic change, just that I can't travel without my child. If I leave the house and it's getting late, I have to hurry and come home. If it happens that where I'm going I can't come back, I go with my child so we have the drugs with us. Because of my child, I can't go to work that would take me out of town where I can't come home. She takes her drugs at 8 o'clock, so when I'm out and it's about 8, I have to leave what I'm doing and rush home. That's my problem.

[Participant 5]

I do not have any problem because I live with her and my mom, so even if I'm not around, my mom will make sure she takes her drugs.

[Participant 1]

I also don't have a problem because my big sister is around, so if I go to work, she takes care of her. Also, her teacher supports. She takes her drugs to school, and her teacher knows of her diagnosis.

[Moderator]

You mentioned that at times when you see your sister isolate herself and become sad, if you see the child in that state, how do you feel as a mother, sibling, or grandmother?

[Participant 2]

When I see it like that, I panic. Even when my child wasn't aware of her diagnosis, she has been like that, so I wrap my hands around her and inquire. She says she doesn't like the food, and I tell her that she should eat that and I will make a better one the next day. When I see her, I worry. Even if tears well up, I hide it, then I use stories to cheer her up. My child asks when she will stop taking the drugs, and I encourage her to continue till the doctors tell her to stop. I find it hard encouraging my child, so I use the Bible to encourage and teach my child. I had difficulties when my child didn't know, but now that my child knows, the pressure on me has reduced.

[Participant 3]

When I see my child isolate, I control myself so I don't show my emotions. It makes me think and worry a lot.

[Participant 4]

There are times my child isolates herself such that even if you speak to her, she wouldn't respond. But she loves songs, so I get her to sit with me, then we sing some—even some kindergarten songs. Her siblings would come around, then it relieves her. I get sad later on. At times, she would come ask if someone is owing me.

[Participant 5]

As I said, when I see her isolate, I want her to be busy, so I engage her. For instance, I ask her to go charge my phone because I know she will definitely go fidget with it. But then, at the end of the day, I still feel bad because we are all human, and she has a future ahead of her. How is it going to be like? But at the end of the day, it's just about encouragement.

[Moderator]

As a caregiver, what difficulties do you face in trying to provide emotional support for your child, because you are not professionally trained to do that?

[Participant 5]

Sometimes you put your happiness aside so the child can be happy. You sacrifice a lot. For instance, if you have GH₵10 and your child wants something, you have to get what the child wants first. That way, no one will get to deceive and do it for your child, putting the child at risk, or the child feels he or she is being discriminated against because of the virus. So it comes with a whole lot of sacrifices.

[Participant 4]

For things like this, I feel they are bound to happen because my older children don't carry the virus, but because of the death of their father, at times they also feel sad, especially when they come from school and they need some school supplies. For that, even if I have to borrow, I do, just to make them happy. Last Sunday was my child's birthday. I didn't have money. She came to me saying, 'Ma, you know today is my birthday.' I replied that I had forgotten—meanwhile I had not, I just didn't have money, so I told her so. Not long, I saw that she had isolated herself, so I thought to myself on what to do. There and then, I called a friend to borrow me GH₵100. Once I received the money, I called my child and we went out to get biscuits and other things, which she came to share amongst her friends. So when it happens like that, I try to figure out why. If she needs something, then I get whatever it is for her. There are times I don't plan on going out, but because of her and the way she isolates sometimes, I just dress up and take her with me on a stroll, and buy her something. By the time we get back, she has been refreshed.

[Moderator]

Has it brought any challenge to you?

[Participant 4]

Yes, because even if you don't have the money, you still have to get what she wants. Sometimes when we prepare banku with pepper, she refuses to eat the pepper, and truly when she sits with us, she can't eat. So I have to prepare okra stew for her. Initially, she doesn't eat banku or kenkey, so when we're all eating banku, I have to pound fufu for her. If you don't do it for her, she won't eat, and if she doesn't eat, it brings another set of problems. So that's my challenge.

[Participant 3]

The challenges are a lot. As I said earlier, even if you don't have, you have to get for the child. Because if the child doesn't eat, that also comes with another problem, so you just accept that it's your responsibility because help comes from nowhere.

[Participant 2]

It's similar to the others. The child's feeding, refusing some meals, and the acting out have become my problem. Just like the lady said her child refuses banku, I face the same issue, so I have to pamper my child. Sometimes the request is to buy yogurt before the food will be eaten because I let her know that I don't have the money to buy another food. Even when my child wasn't aware, I had to spoon-feed.

[Moderator]

Has the knowledge of your child's diagnosis brought any changes in the relationship between family members? For instance, how the other siblings and family who know—has it changed the way they relate?

[Participant 2]

For me, no one except my child and I knows, so there hasn't been any change in our relationship.

[Participant 1]

For me, I go to work, so I'm not always at home. But when I get back, she comes to me saying she had eaten, so what will I eat? I tell her that I have eaten, so she is happy anytime I get home.

[Participant 3]

Initially, my child sleeps together with my sibling's child, but I don't know if it's because of my child's weight loss—now when my child enters their room, they send my child away. It's my sibling's child. Because of that, my child has also stopped going close to them.

[Moderator]

But your sibling doesn't know of the diagnosis?

[Participant 3]

No, they don't know. I'm the only one who knows.

[Participant 4]

No one in the family knows, but aside from the things on her skin which caused other kids not to play with her, no one knows—even her older siblings. I haven't told them. Sometime ago, her elder sibling came back from school. He's the only one I told recently because I don't know when I will sleep and not wake up, and I'm the only one who comes for her drugs. So when my eldest child came home, I told him, 'This is what is going on with your sister, so in case you come home one day and I'm not around, know that your sister is on drugs.' I'm not telling you so you hate your sister. He told me that it's not a matter of hate, that I shouldn't tell anyone. So it's just my son I told about it, so no one knows.

[Moderator]

So your relations and interactions in the house have not changed?

[Participant 4]

No, they haven't.

[Participant 5]

For me, our relationship has not changed, and no one knows about her diagnosis because I live with just my mom and siblings. As for my mom, I'm the only one who knows, but when my sister got sick, she had a cough. When the doctor saw her tongue, he requested labs. I wasn't there by then—it was my kid brother who was, so he is also aware now. But he has been spoken to, so he is okay. He doesn't even live here; he's at Kaneshie. So it's just him, my mom, and I who know about it.

[Moderator]

So it has not affected the relationship in the family?

[Participant 5]

No, no, no. From the beginning when I cook, she helps me out, and till now we still cook together.

[Moderator]

Assuming this hospital wants to be of help to you, what form of help or support do you think will help you deal with the pain and emotional stress that comes with caring for adolescents living with HIV? What help or support do you feel the government, a hospital, or an NGO can provide to help you cope with the pain and emotional stress, and help improve the relationship between you and the child and other family members so you have some relief?

[Participant 4]

For me, the support that will be helpful is that at times I don't have money to finance her feeding and other necessities, but I have a shop. If they should give me financial support to fill my shop with goods, I think when I sell, I will be able to provide for her needs.

[Participant 5]

My business—I sell panties and perfume. Should I get capital to support, when she needs something, everything will be okay. Even though right now everything is okay, as time goes on because she is growing, her needs keep expanding. If I get support with my business, I will be okay.

[Participant 3]

I need support with my business. I sell fairly used clothes, so if I get some money to support it, whereby if the child needs something, I can easily provide.

[Moderator]

How about counseling? Should someone want to offer counseling so that both you and the child can overcome the pain and emotional stress you go through, and the child would be able to face the virus head-on? Apart from the economic support, which is well noted, which other support will help you overcome the emotional challenges you go through?

[Participant 5]

Should we get an organization, like a youth wing, where once in a while they will meet and have someone who will speak to them, where they play games like ludo. So all the time they know they are not alone—that is also not bad.

[Participant 2]

For me, at my age, I don't know any business or work I can venture in which I will be able to do, so I would like them to support my child in her education and other things so my child can have a future.

[Moderator]

Is that the only support you think will help you?

[Participant 2]

I don't have the strength to work now. Should I be given capital to work, I don't have the strength to do so, so they should support my child in her education and other things.

[Moderator]

And the support that will help her overcome the emotional stress she goes through—what support do you think your child needs that will help your child face the virus head-on, a support that cushions the child so there is no emotional stress? When the child overcomes all the emotional challenges, you as a caregiver also have peace of mind. As for the economic support, I understand, but the support that you know will help the children to be independent enough to take care of themselves with no emotional challenges.

[Participant 4]

Just as the sister said, maybe once in a while, just like we have come, we should engage in games or play. When the child comes, they meet other children. That encourages them that they are not alone because sometimes they think that they are the only ones with the virus. Should we get such programs, like games—ludo—where they meet like once a week or once a month...

[Participant 5]

...or maybe during vacation, Christmas holidays, or the Easter holidays.

[Participant 3]

Just like they are saying, a program that brings the adolescents together, like on a day they are not in school, doing activities like this would help.

**Introduction of Multiple Family Group Therapy (MFGT)**

[Moderator]

Before we continue with the discussion, I would like to share something with you on why we are here at Atua Government Hospital. We want to initiate a program called Multiple Family Group Therapy, and what the program does is bring together different families—like sister and her junior sister, mama and her child, you and your child, grandma and her grandchild—once every week to have interactions. We will train health workers to lead you and teach you how to handle stress, how to solve problems that arise in the family, how to provide support for your child should he or she be going through emotional stress, how as a caregiver you help the child overcome it, how to have healthy interactions and relations with your child, not undermining the child but listening to them to give them a sense of belonging, how to manage the child's health and wellbeing, how to help the child cope with stigma or discrimination, and how to empower the adolescent mentally and emotionally so they can stand on their own till they grow.

This is the program we intend to introduce to you. We bring about 8-10 families together. We have lots of topics to deal with—how to deal with stress. We want to include you in planning and organizing the program, so I will continue to ask you a few questions to take your thoughts on how well to plan and organize the program so it is successful. For the fact that we are inviting you, your transportation will be taken care of.

[Moderator]

Which day of the week would be convenient for you? You are to come with your adolescent, so consider school.

[Participant 4]

Wednesday.

[Participant 5]

Yes, school. For my sister, she is in JHS 3 and they write mocks every now and then, so I had her take permission from the school yesterday so we can be here today. When we go back home, she will go for extra classes. On Saturdays too, maybe you have an engagement and other house chores, so Sunday after church.

[Participant 3]

I would say Saturday because of their education. With Sunday, not everyone can make it.

[Participant 2]

Sunday will help me, after church.

[Participant 1]

Sunday after church.

[Moderator]

I know the children are in school now. When are they going on vacation so we can schedule for then? Madam said she would have preferred Wednesday, you also said Saturday, and three people said Sunday. During vacation, we know that they are home, so for any day we decide, they can make it. Will Sunday after church not help?

[Participant 5]

Please, will it be every week or once in a while?

[Moderator]

Once a week for fourteen weeks. When we come, we will spend 2 hours with lots of activities that will be very interesting, just as you suggested. Is Sunday after church okay? What time after church—say 1 o'clock? Will Sunday help?

[Participant 5]

Yes, because churches close early now. When we come at 1, by 3-4 we are out of here.

[Participant 4]

For me, Sunday would not help because I have meetings at church after we close, but I would get a replacement.

[Moderator]

But you are the one we would be needing here.

[Participant 4]

I mean someone to stand in for me at church.

[Moderator]

Good, God bless you. So for the fourteen weeks, you get a replacement at church. So Sunday after church, 2 hours. Are you okay with that? What support can we give to you to ensure that every Sunday after church you would be here for the 14 weeks without excuses? Be open. We want you to come because we have realized that the moment a child is diagnosed with the virus, they grow with mental problems—some with depression, anxiety—and without help, it becomes mental instability or madness. We want to teach you how to relate with the adolescent so that as they grow, they can overcome all the mental and emotional problems. They'll grow with a strong mental capacity and can adhere to their drugs and take care of themselves even in your absence. What support should we give to you to ensure that you would come? One thing is transportation.

[Participant 2]

I feel that when it's Sunday, you call us. Personally, I forget easily, so call us.

[Participant 3]

Just as you said, transportation. Maybe it will get to Sunday and you are financially challenged, so the transportation would help.

[Moderator]

We want to make sure that you would come. We want you to say it so when it gets to the time, you wouldn't have any excuse not to come. So mama said we should call and remind you, and madam also said transportation. Is that all?

[Moderator]

What practical arrangements would make it easier to participate? Our sitting arrangement, how the discussion would go—maybe if you want the health worker to teach you, or we do it as a discussion so everyone can share their views on a topic, or we dramatize or role-play a topic like communication after discussing it. What arrangements should we employ that will make the program a success? Like where we would meet for the program, whether we should meet as a group or at times meet individually.

[Participant 4]

If it were up to me, I think we should meet as a group because with some of the experiences, someone would share theirs and another would learn from it, so I think meeting as a group is the best option.

[Participant 3]

I agree with what Participant 4 said. Maybe this sister says something I must have not heard before, and I will also pick a lesson—maybe how I will care for my child from picking up lessons from what another will say.

[Moderator]

Apart from letting the health workers lead you, is there anything else you think we can do so you can benefit? Should we show some videos, display a drama, or role-play?

[Participant 4]

Please, if there is a video you can show us. Because when I found out about my child's diagnosis, I was pained. At the facility where we take the drugs, after the second visit, I heard in town where someone said they saw me at this place, so I stopped going for the drugs altogether. One day I was watching television when a lady said that she has also had the diagnosis and been on drugs for 40 years, but there is nothing to show that she is a carrier. When I looked at the woman who also has the virus, I realized that I was making a grave mistake, so I would go for the drugs for my child again. If the woman has been taking the drugs for 40 years and she is fine, then my child too would grow and clock 40 and above too. So if there is a video you can show, maybe there is someone who does not have faith in the drugs. If there is a video that would encourage them so the person can get the interest to take the drugs, it would be nice.

[Participant 5]

What she said is true because from the onset, I went on TikTok to search for videos which I showed to my sister. You see the carriers who are bold enough—when you go on TikTok, even when they go to work, the drugs are with them where they explain and demonstrate how they take their drugs. Should you show the videos at the meetings, it would help. Also, the environment we would meet shouldn't be an open place where other people would hear us.

[Moderator]

So privacy and confidentiality. Do you agree?

[Participant 2]

Yes.

[Participant 1]

Yes, I agree with her.

[Moderator]

So in sum, you are saying that we should get a place where our discussion would be private. Where do you want us to have these meetings? Should it be here at the hospital where we would get a room only for our discussion? Are you comfortable that we have the meeting here at the hospital?

[Participant 5]

Yes.

[Participant 1]

Yes, I'm comfortable.

[Participant 2]

I'm comfortable.

**[Moderator]**

What kind of topics or issues do you think should be addressed in the program sessions?

[Participant 5]

I believe that even though our meeting is all about HIV, it shouldn't be just that. It can be lifestyle—the way the adolescent would dress, how to carry themselves, bathing, personal hygiene—should also be included so the meeting would not be only about HIV. In the house too, we talk about how they care for themselves as HIV carriers, so we can include other topics like their diet and other things.

[Participant 4]

Just as my sister said, if you could include other topics that would help in our lifestyle. Sometimes in life, you would be doing something thinking that it's the right or correct thing to do, but someone would say or share their experience or thoughts on the matter, which would help you realize that you can adapt some of their ideas. So if you have other topics, our meetings wouldn't only be based on HIV—it would help.

[Participant 3]

They have said it all.

[Participant 2]

When we come, just as the others say, let's add the word of God because it would help us get hope.

[Moderator]

In the beginning of the discussion, you said that the child had a rash breakout on the skin, and because of that, people didn't want to mingle with them. Sometimes the children themselves didn't want to also go near others, isolating themselves. This is called stigma. If we discuss it—how to boldly face stigma and mingle with others—would you like that?

[Participant 5]

It's very important because they are not always at home. They will go to school, church, and other gatherings where they will meet other people, so they need to be brave enough to face people and overcome stigma. There are people who would always want to bring you down because of your diagnosis. Maybe at school, for instance, my sister is really smart at school, but there would be another child who would tease her because of her condition, so the stigma topic is very important. It's not always we would be with them. They would go to school; you would send them on errands where they would meet people. If you teach that, it will help.

[Moderator]

What would prevent you from coming for the program, or when you come, would hinder you from participating regularly? I would want us to solve it now. If we wait till we begin the program, solving these hindrances would be difficult. What challenges might prevent your participation regularly?

[Participant 5]

For now, I can't say, but there are emergencies that would put you in a tight spot—maybe you need to rush someone to the hospital. I mean serious emergencies. Aside from that, I would be able to come.

[Participant 4]

My issue is what I made mention of before, and I would be able to sort it out. I will get someone to stand in for me at the church meetings. Aside from that too, as my sister said, if there is no emergency, I'm ready to come.

[Participant 3]

Nothing will hinder me unless it is an emergency where my attention would be needed, like funerals or when someone dies and I would be called to come. With family members, everyone is fighting for themselves, and everyone has their life to live. My child's life is very important to me, so I would come.

[Participant 2]

For Sunday, if an emergency case does not call for my attention, I will come. My issue is with my child. I find it difficult bringing her to the hospital. Even coming for this meeting was hard bringing her, so when the meeting begins and I ask her to follow and she agrees, I will come.

[Moderator]

With what you are saying, what do we do to sort that challenge out?

[Participant 2]

I would like you to speak with her to get an understanding so she would be willing and comfortable coming for this meeting.

[Participant 1]

For me, once I don't have anywhere to go that Sunday—if my brothers or mother do not call me to come and help with an emergency—I will be able to come.

[Moderator]

Would you prefer a health worker to lead you, or sometimes we would pick one of you caregivers to lead? Like for some of the topics which are more family-oriented, we would pick one of you caregivers with experience to lead that day's discussion. I told you we are doing this with you, so share your thoughts with me so it will be successful and coming here will be worthwhile for you.

[Participant 3]

Please, that will also help. When a caregiver shares their thoughts, others would also share their experience, then we learn.

[Participant 5]

It would be nice to have the health workers to lead and also sometimes a caregiver, because there are some with a lot of experience. I have been doing this for just 5 months, but someone has been doing it for 8 years, 5 years, 1 year, who could also teach. We also have to engage the adolescents because sometimes when they go to school and some programs, they are very active, but sometimes we underrate them, thinking they know nothing. They experience some things which they may not be able to tell us but can tell the nurse, so we have to engage them so they can ask all the questions they have in mind.

[Moderator]

After our discussion, we would meet with the adolescents so they also share their thoughts. We then put everything together and analyze it.

[Moderator]

Is there any additional recommendation you would make, or anything you want to add that I did not touch on?

[Participant 5]

Yes. During your discussion with them, I would like you to let them know that there are no drugs apart from the ones they are taking that will help them. For now, I don't know about any drug aside from the ones people have been advertising. Maybe the child has discussed their condition with another person without our knowledge, then the person discourages the child from taking the ARVs but says another will cure them, thereby making them neglect the drugs that will help them. So they have to be aware that for now, this is the only drug that will help them.

[Participant 3]

You said you are going to discuss with the adolescents. I haven't disclosed to my child yet.

[Moderator]

Our meetings will be based mainly on HIV, so if the child doesn't know and all of a sudden they come for such a meeting where all they hear is HIV but the child is not aware of his or her diagnosis, it wouldn't be fair on the child. So with the kids, we want those who know their diagnosis. We would register you when we are done with the discussion, so if by the time we begin the meetings you are able to disclose to your child, we would call both you and your child.

[Participant 2]

This issue happened to my child. She wasn't aware when a program was called here, and it was disclosed to my child—we had planned to tell her later. That day it disturbed my child a lot. So I told Mr. Omari that my child didn't know. He apologized and called my child aside and spoke with her, but upon getting home, I realized that my child was still shaken.

[Moderator]

You see, so that is why we do not want to involve such a child, where at the meeting we keep mentioning HIV. In any case, from now till March ending, if you are able to disclose to your child, I will give out my number so you call and let me know. From the day you disclose to the child, you monitor them to see if there are any changes in their behavior and emotions.

Also, from today, any changes you see in the child's behavior, let us know because we are very interested in their mental health. If the child does not have a strong mental capacity and health, the child would have challenges relating to others, also feeling inferior to others. The child's confidence level will reduce, and if that happens, it affects their education. So the mental health of the children is very important to us, and if they do not have good mental health, they will not take the drugs.

Let us know any changes in their behavior that we will note. This will help us know how and what to discuss during the meetings—the teaching and training we will give to strengthen their mental health. Raising a child with strong mental health, you are assured and confident that your child can care for themselves in your absence without worries. Be observant and report any changes to me.

END OF TRANSCRIPT
