# Supplementary material for: Potential enablers for the implementation of multiple family group therapy intervention in the lower Manya Krobo District, Ghana: Perspectives of multiple stakeholders
Source: PLOS Glob Public Health. 2026 Jan 16;6(1):e0005620. doi: 10.1371/journal.pgph.0005620 (PMC12810799; doi:10.1371/journal.pgph.0005620)
Supplement: S3 Data — (DOCX) [file pgph.0005620.s007.docx]

**Individual Interview Transcript**

**Adolescent Living with HIV**

INTRODUCTION AND CONSENT

[Interviewer]

I'll conduct an interview with you. I'll be asking you a few questions. It is just between you and me. You can trust me and share your deepest feelings, secrets, and information with me. You don't need to hide anything because everything discussed between you and me stays between you and me. Okay? It won't go any where. Even if it is published, your identity will be anonymized.

[Respondent]

Okay.

[Interviewer]

Would you want me to speak English throughout, or maybe we can mix it with Twi?

[Respondent]

Yes, Twi and English.

# DEMOGRAPHIC INFORMATION

[Interviewer]

What's your age?

[Respondent]

16.

[Interviewer]

Are you male or female?

[Respondent]

I'm female.

[Interviewer]

What grade level are you? I know you are in school.

[Respondent]

Grade 8.

[Interviewer]

Is it secondary school or JSS?

[Respondent]

It's JSS.

[Interviewer]

So grade 8 is JSS 2?

[Respondent]

JSS 2.

[Interviewer]

Great. Thank you. How long have you been living with HIV, if I may ask?

[Respondent]

One year.

[Interviewer]

Which means you were only told one year ago?

[Respondent]

Yes.

[Interviewer]

But when mommy told you, did mommy tell you that you got it at birth? That when you were born you already had it?

[Respondent]

She didn't even tell me that I was having the disease.

[Interviewer]

Okay, mommy never told you that you were having the disease?

[Respondent]

I found it myself.

[Interviewer]

You found it yourself? How? How did you find it yourself?

[Respondent]

She told me to go and clean up her room. The drugs that I've been taking have a label, so when I was going to take the drug and I saw the label, they had written on it that 'HIV is real.'

[Interviewer]

Okay. That's so smart of you. So you only found out one year ago, but you don't know for how long you've been living with the disease, right?

[Respondent]

Yes.

## Living Arrangements

[Interviewer]

Who do you currently live with? Do you live with both parents, an uncle, a sister, or your whole family?

[Respondent]

My aunty, but I don't live with her anymore.

[Interviewer]

So who are you living with now?

[Respondent]

My brothers.

[Interviewer]

How many brothers do you have?

[Respondent]

Five.

[Interviewer]

Do you live with all five brothers in the same room?

[Respondent]

No, two brothers.

[Interviewer]

Where you live, is it a rented apartment or a family house?

[Respondent]

My brothers have been saying that it's a family house.

[Interviewer]

Is any of your brothers married? The brother that takes care of you, is he married?

[Respondent]

None of them is married.

**MAIN QUESTIONS**

[Interviewer]

I want to find out from you—and I just want you to be frank and sincere with me, okay—because I am interested in how you are coping with the disease and how HIV is affecting your academics and even your mental health. So just be frank and sincere with me because I understand your situation. Okay?

[Respondent]

Okay.

[Interviewer]

How would you describe your emotional well-being since you discovered you have HIV? Has it had any effect on how you think? Has there been times that you feel so sad? Times when you feel like 'I wish I wasn't born' or 'I wish I was dead'? Do you feel anxious that something can happen to you? Have you had such experiences ever since you found out you have HIV? Share some of these experiences with me.

[Respondent]

Yes. When I found out that I was having HIV, they admitted me to the hospital for 3 weeks, and I was sleeping there. When I was discharged, I went home. I told my mom, 'So you people know that I am having this disease, and you didn't tell me.' They asked me how I knew, and I said I found it on the label. Then I started crying. In the night when everybody was asleep, I even wanted to kill myself because I couldn't breathe. Then my aunty's son, who is the eldest, came and told me it doesn't mean it's the end of my life. He took the knife from me and told me to go and sleep. At times, I feel like, 'Am I going to leave this world whilst I have not completed my education?'

[Interviewer]

Are there times that you get so sad, so worried? Have you had such experiences where you're always thinking about the disease, always worried about what people will say or think about you?

[Respondent]

Yes. When I was with my aunty, the reason why she brought me back was that a pastor came to our house when I was in the hospital, so I didn't know. When I came, they told me. They said the pastor said that for me, someone gave me the disease, so I'm a witch. They say I'm the reason why my aunty is not married. They say I want to snatch all my aunty's money. So after I was discharged to the house, my aunty was querying me. She said, 'So I want to destroy her life.' And she brought me back. She even sent me to the village where none of my family members were there, and my brothers came for me.

[Interviewer]

Sorry. I understand how you feel. But are there other times that you have fears about where this disease is taking you? Especially because you've heard that with this disease there is no cure—you have to be on the medications for life. Do you have any fears? Tell me some.

[Respondent]

When I was in Accra, we read about HIV. After closing, I asked a friend, 'Can anyone who is having HIV/AIDS be alive for so many years?' What she told me was, 'If you drink the medicine, you'll be good, but still you will die because the disease will not go forever.' So at times if I'm there, I will be feeling like not drinking the medicine because by all means I'll die.

[Interviewer]

How has this affected your academics? You getting to know that you have HIV—has it had any effect on your learning, you going to school every day, you relating with your peers in school? Has it had any effect on your academics, your normal activities, or even your relationship with your peers, colleagues, or friends in school?

[Respondent]

Since I found out about the disease, I don't even like going to school anymore. I don't really talk to my friends. Since last week, I didn't even go to school because of how my sister has been reacting.

[Interviewer]

Your sister?

[Respondent]

She talks to plenty of people, so she went to tell her friends. Anytime I'm passing, they have been watching me. I don't even want to go to school again.

[Interviewer]

Are you comfortable playing with your mates and your peers both at home and in school?

[Respondent]

No, I don't play with them.

[Interviewer]

Why? They don't know you have the condition, so why are you not playing with them?

[Respondent]

I know, but my brother said I should not go out. So every day I'm in the room.

[Interviewer]

Do you feel that sometimes people gossip about you because they think maybe you have the disease?

[Respondent]

Yes. My best friend and my sister's daughter.

[Interviewer]

Do you hear some of the things they say about you? What do they say?

[Respondent]

Sometimes, if they are talking and they see that I'm coming, they just keep quiet and just be laughing.

[Interviewer]

But you don't hear what they say about you, right?

[Respondent]

No.

[Interviewer]

Is it part of the reason you have withdrawn from them and you are always indoors? Is it because of the gossip?

[Respondent]

It's because of their attitude. My brother said that I should not follow them because every evening they'll go out and they'll come at midnight. At first when I came here, I didn't know that that's what they have been doing, so I followed them one day. When we came back, they said that if we go home and they ask us, we should say that we went to fetch water. Me too, I didn't lie. I said the truth. That's when they began to gossip about me.

[Interviewer]

You are so strong. You are a strong girl, okay? How do you cope with all these things you are telling me—the extreme sadness, people gossiping and laughing at you, not having people to play with, becoming so sorrowful that sometimes you feel like you need to even kill yourself? How do you cope with all these things on your own? What do you do?

[Respondent]

I always take my mom's picture. My mom is no more. I will be watching her picture every day if I am sad. It makes me happy.

[Interviewer]

So do you find or get inspiration from just looking at your mommy's picture?

[Respondent]

And the Bible, because my class teacher has been coming to me to read the Bible.

[Interviewer]

So who do you get to talk to when you are sad? Do you have anybody that talks to you, that encourages you, that comforts you sometimes? Is there anybody doing this?

[Respondent]

No.

[Interviewer]

So you don't have anybody to talk to and share your deepest pains, share your fears with? You have nobody to talk to?

[Respondent]

No.

[Interviewer]

Do you also resort to prayer when you are sad?

[Respondent]

Yes.

[Interviewer]

How do you do this? Is it you praying on your own in the room, or you go to the church and pray with other people in a group?

[Respondent]

No. I just stay in the room and lock myself and pray so that no one can hear me.

[Interviewer]

You pray silently?

[Respondent]

Yes.

[Interviewer]

What about going to church? Do you go to church often?

[Respondent]

No, I don't go to church often.

[Interviewer]

Why don't you go to church?

[Respondent]

My brothers have been going to church with me, but now they don't want to go to church again.

[Interviewer]

Your brothers don't want to go to church again, so you have also decided to join your brothers?

[Respondent]

No, I don't know the way to the church.

[Interviewer]

In your neighborhood, do you get any kind of support from anybody? Maybe financial support, nutritional support—maybe once in a while somebody buys food for you? Somebody who, if you cannot go for the drugs, will go for the drugs for you? Somebody to talk to you, comfort you, and make you feel calm? Do you get any support like that?

[Respondent]

That's my big brother.

[Interviewer]

Your big brother goes for the drugs for you?

[Respondent]

No, he comforts me. I have never seen him before, but I've been speaking to him on the telephone.

[Interviewer]

Who is that person you have been speaking to on the telephone?

[Respondent]

Emmanuel.

[Interviewer]

Who is Emmanuel?

[Respondent]

I'm after him. I'm the last born.

[Interviewer]

And Emmanuel is your senior brother? He is the fifth?

[Respondent]

Yes.

[Interviewer]

Of all the people in the family, why do you find Emmanuel as the best person to talk to when you are sad? Why not any other person in the family?

[Respondent]

It is because he is my real brother—same mother, same father.

[Interviewer]

So you are more attached to Emmanuel than the other brothers?

[Respondent]

Yes.

[Interviewer]

Did you and Emmanuel grow up together?

[Respondent]

No.

[Interviewer]

Apart from your brothers going for the drugs for you and you talking to Emmanuel to encourage and comfort you in your moments of sorrow and sadness, what other supports do you think would help you? Assuming somebody wants to help you and provide you with any needed support, what kind of support do you think would be so helpful to you, especially to deal with the emotional pain, the sorrow, the sadness, the weeping? Which other support do you think will help you especially deal with the emotional challenges and overcome the pain and sorrow of living with HIV/AIDS?

[Respondent]

I need someone to help me with my education.

[Interviewer]

Okay, somebody to help you with your education, like maybe a scholarship? Somebody that will help pay your fees? What else?

[Respondent]

My brothers have been traveling often. I don't have a telephone. They say they will buy it for me, and I said I don't like it. I don't like the phone. I want something like a laptop that I can use to learn because if I am holding a phone, the boys will be calling me, and I don't like that.

[Interviewer]

You said you want a laptop? But how would a laptop help you deal with the crying, the sadness, and the sorrow?

[Respondent]

I'll watch movies.

[Interviewer]

Does that mean when you watch movies you feel okay?

[Respondent]

Yes, but in the house we don't have television.

[Interviewer]

You have mentioned educational support and a laptop for recreational activities like watching movies. What else? I am particularly concerned about the emotional problems you have—the fear about death, having suicidal thoughts sometimes, getting extreme sadness, depression. What can we do to help you overcome these things? I am particularly concerned about these things because if you have mental stability, you can navigate life successfully no matter what your situation is. So what can we do to help stabilize your mind so that in spite of the HIV, in spite of the gossip, in spite of people laughing at you and pointing fingers at you, you are still stable in your mind? You don't care. Are you getting what I am saying?

[Respondent]

Yes.

[Interviewer]

You are stable in your mind. You don't care, and you are able to live this life successfully and grow into your old age. Has anything ever occurred to you that when I get this kind of support, I will be stable in my mind so that in spite of my condition, in spite of people laughing at me, in spite of people gossiping about me, I am a strong girl or I will be a strong woman? Has anything ever come up in your mind that when I get this kind of support, I will be fine?

[Respondent]

Yeah. I already told you that the pastor said I am a witch, and he is still saying it. So I want somebody to take me back to Accra so that I will go and tell the pastor that I'm not a witch, so he should stop saying that.

[Interviewer]

So you need us to speak to the pastor so that the pastor will stop saying that you are a witch?

[Respondent]

Yes.

[Interviewer]

Let me encourage you, my dear. You are not a witch. Okay? You are a child of God. You were made or created in the image and likeness of God, so you are God. Okay? Have this kind of mentality. Are you getting what I am saying? One, you are a child of God. Two, you have been created in the image and likeness of God, so you are God. Can anybody trample on God?

[Respondent]

No.

[Interviewer]

If you have this kind of mentality, you feel superior. Nobody can bring you down. There is nothing that anybody says that will dim your light. You are a light just as God is, so are you. Forget about what the pastor is saying. Forget about anybody trying to bring you down or trying to destroy your self-image. Anytime you are walking, see yourself as a child of God and therefore God. Are you getting what I am saying?

[Respondent]

Yes. I understand but I still need you to tell the pastor I’m not a witch. My family don’t like me and doesn’t treat me well because of what the pastor said.

[Interviewer]

Forget about anybody calling you names—'you are not beautiful,' 'you are a witch'—forget it. They are all lies from the pit of hell. What is true and what you must believe is what the word of God says about you. Are you getting what I'm saying? So make the Bible your friend. Read the Bible. You can only believe what is true, and what is true is what the Bible says. Every other thing anybody says is false, and you cannot believe falsehood. Are you getting me?

[Respondent]

Yes I do.i will try and stick to the advice but its not easy …….sobbing.

**[Interviewer calms her and refer her to the psychologist]**

**Interview continues the next day**

INTRODUCTION OF MULTIPLE FAMILY GROUP THERAPY

[Interviewer]

How are you my dear. I hope you had a great time with the psychologist.

**[Respondent]**

Yes please.

**[Interviewer]**

I hope you are ok so can we continue with the interview

**[Respondent]**

Yes please

**[Interviewer]**

Ok, we will continue from where we left of yesterday. This brings me to why we want to come to Atua and engage you and your caregiver—engage you and your brothers. We want to come to Atua and organize people like you and your brothers or your families. We want to bring you together and speak to you about dealing with HIV, dealing with stigma, dealing with the emotional challenges that you go through. We also want to speak to you about how you and your family can live cordially without responding to name-calling, without responding to what people think and say about you. How you can deal with stress, how you can comfort yourself, how you can cope with all the negative challenges of living with HIV/AIDS, how you can receive support and give support to anybody in your family. Are you getting what I am saying?

[Respondent]

Yes.

[Interviewer]

The name of the support my team and I are bringing to you is called Multiple Family Group Therapy. We are calling it Multiple Family Group Therapy because we are bringing different families together so that we can help you share your experiences with each other—share the challenges that you go through, just as you are sharing with me. Then we are going to train health workers. I'll also be at the forefront assisting you. We are going to train health workers so that they can deliver training to you, empower you mentally, empower you emotionally so that you'll be able to deal with all these things. Okay?

[Respondent]

Yes, please.

The laptop you need is a secondary matter. The education is a secondary matter. All these things will follow. The Bible says, 'Seek ye the kingdom of God first, and all these things will follow.' Today I want to tell you: seek things that strengthen your mental health. Seek things that empower you to deal with any negativity that comes with living with HIV, and every other thing like the educational support you need, the laptop you need, the money you need—all these things will follow because your greatest asset in this life is your mind. Your greatest asset is not money. Your greatest asset in this life is your mind, and that is why my team and I are coming to engage you and empower you so that you become mentally strong and emotionally strong.

[Respondent]

Yes Please, Thank you.

[Interviewer]

Excellent. So this is the program my team and I want to introduce to you in Atua. The name of the program is Multiple Family Group Therapy. We bring different families with similar challenges to share experiences, learn and support each other. I am particularly interested in your mental health because your greatest asset in this life is your mindset. Are you getting what I'm saying?

[Respondent]

Yes Please. I do.

**[Interviewer]**

Are you willing to be a part of this program.

**[Respondent]**

Yes please

**[Interviewer]**

We'll be organizing this program in Atua. Because we are doing this program for you, I want us to continue with the interview and find out how you want this program to be organized to suit your own preferences because we are doing it for you. The program is meant to benefit you. So I want to solicit your ideas on how this program can be organized successfully to suit you and your family or your own preference. It is what you will tell me that is what we will do, because at the end of the day, if we are imposing on you, we are not going to achieve anything. I want you to be sincere, be frank with me, and tell me how you want the program to be organized so that it can suit you and help build you up.

**[Respondent]**

Ok, I am ready

[Interviewer]

Assuming we are organizing the program, which day and time will be favorable for you so that you can participate every time? Which day will be favorable? I know you are a student—you sometimes go to school from Monday to Sunday. On which day will it be favorable for us to bring you and your peers and your parents or your siblings together once a week to have an interaction or a discussion with you about your mental health and the challenges that you go through?

[Respondent]

Saturday.

[Interviewer]

Are you sure Saturday will be favorable for you? What about your brothers, because when you are coming, you'll be coming with one of your brothers?

[Respondent]

Yes, one of my brothers does not go to work on Saturday.

[Interviewer]

What about Sunday?

[Respondent]

On Sundays, none of them go to work.

[Interviewer]

So that means we can put it on Sunday so that the other mothers who go to the market on Saturday will be able to join us, isn't it? Do you think Sunday will be a great day for us to have these meetings?

[Respondent]

Yes.

[Interviewer]

I know you go to church sometimes, so would you prefer after church? Be frank with me.

[Respondent]

Yes.

[Interviewer]

You want it after church, right? After church, what specific time?

[Respondent]

3 pm.

[Interviewer]

3 pm on Sunday. If you have to be coming for the program, what support can we give you that will make you come regularly so that you would not have any excuse and say, 'Oh, today I couldn't come,' and then Auntie Helen will call you and you will say you didn't have transportation? What support can we give you to ensure you can come for the program until the program is over?

[Respondent]

Transportation fare and food.

**[Interviewer]**

Are you sure that is all you will need

**[Respondent]**

Yes, For now Yes

[Interviewer]

Are you comfortable having meetings which are made up of you and other adolescents together with your parents? Are you comfortable having discussions with your peers and your parents or your siblings inclusive?

[Respondent]

No, only my peers.

[Interviewer]

So you are comfortable having discussions with only your peers?

[Respondent]

Yes. Only my peers

[Interviewer]

What about discussions that talk about how parents should treat their children, how parents should talk to their children, how parents have to listen to their children? Do you think that discussions that focus on the family and how to take care of an adolescent with HIV—we can bring you, the adolescent, and your parents or your siblings together?

[Respondent]

Yes. For discussion about the family and how they take care of us adolescents, we can mingle with them.

[Interviewer]

Those ones you'll be comfortable for us to bring you people together. But what kind of discussions would you not want to share such information in the midst of the parents, but you are comfortable sharing such information with only your peers? Discussions about which topics, so that when we are structuring the program, we know that when it comes to discussions about maybe sexually transmitted diseases, we will do it with only adolescents? What kind of topics would you not want us to discuss in the midst of you and other parents or your siblings?

[Respondent]

Discussions about sexuality and how the HIV came about.

[Interviewer]

You don't want such discussions to be made in the midst of you and parents, but you are comfortable to discuss these with only the adolescents present, right? You don't want the parents to be around when we are discussing anything about sexuality, how you got the HIV, and all of that?

[Respondent]

Yes.

**[Interviewer]**

Where would you want this intervention to be held? The meetings. Do you want us to have these meetings in Atua, maybe a space provided in Atua Government Hospital, or you want it conducted maybe within the community, a space in the community?

[Respondent]

I want it in the community.

[Interviewer]

What about in Atua Government Hospital? We will not come to the clinic. We will get a nice space, maybe the conference room, then we will gather all of you there.

[Respondent]

That would be nice.

[Interviewer]

So you think that a space within Atua Government Hospital, for instance the conference room, will be okay for you?

[Respondent]

Yes.

[Interviewer]

Who do you want to deliver the training? Do you want the health workers? Assuming we want somebody to lead discussions about stigma, discussions about sexuality, discussions about healthful communication, who do you want to lead such discussions? Is it the health workers or the parents?

[Respondent]

The health workers.

[Interviewer]

You are comfortable having one of the health workers lead discussions with you, for instance discussions about your sexuality, your sexual patterns, how you got the HIV, and all of that? You are comfortable having a health worker lead or facilitate such discussions with you?

[Respondent]

Yes.

[Interviewer]

In every meeting, how many hours do you think you can manage? When we meet maybe on Sunday for the training, how many hours can you manage for each training on Sunday?

[Respondent]

I don't know.

[Interviewer]

My dear, You need to say something... You can stay for close to how many hours?

[Respondent]

10 hours.

[Interviewer]

Are you sure you can stay for 10 hours in a day?

[Respondent]

Yes.

[Interviewer]

Wow, that's great. 10 hours in a day.

**[Interviewer]**

What other kind of topics do you want us to discuss or talk about when we meet as peers? When we meet as peers—meaning only the adolescents meet—what kind of topics do you want us to discuss, apart from sexuality, your sexual patterns, and how you got the HIV that you've mentioned to me? Which other topics do you want us to discuss when we meet as only adolescents?

[Respondent]

Prevention of HIV.

[Interviewer]

What else? Don't you want us to talk about stigma? Because you said people gossip about you, they point fingers at you. When you are coming, they will stop talking, then all of them will burst into laughter—that is stigma. Don't you think we can discuss something about how you can deal with this stigma?

[Respondent]

I think so. Yes lets talk about stigma during the program because I have been a victim of stigma for so long

[Interviewer]

Great. What else do you want us to discuss? It is what you are telling me that is what we will do. So don't hide anything from me. Everything you are telling me is what we will implement. Be bold, be courageous, and be frank with me. Tell me what else do you want us to discuss when we meet as peers? I too am an adolescent. So when we meet as adolescents, what do you want us to discuss? Some of the things that you think can help you—things that when we discuss, can really help you. Tell me some.

[Respondent]

Growth in adolescents and physical changes in adolescence.

[Interviewer]

What about how to communicate within your family? How to communicate the fears you have? Sometimes you can be going through certain challenges, and you don't even know how to express it. You don't even know how to talk to somebody about it. Sometimes you have somebody like Emmanuel, but you don't even know how to tell Emmanuel that sometimes you get so sorrowful that you can't even sleep. Do you get what I'm saying? There is a way of communicating how you feel to people. Are you getting what I'm saying?

Let me give you an example. For instance, there is this boy in your class you like. You can't even tell the boy, 'Ah, chale, I like you. I like your vibe. I want to be so close to you. I want to become a close pal.' That feeling is there, but you don't even know how to communicate it. Are you getting me?

[Respondent]

Yes. That is very important Because sometimes I feel very shy to communicate I feel. Sometimes I have to hide it and pretend all is well.

**[Interviewer]**

Great! Do you think that we can talk about how to communicate feelings? How to communicate hurts? If I'm hurt, I should be able to communicate that. 'Mommy, the way you spoke to me, I didn't like it.' If you go out and somebody touches your bum, you should be able to tell the person. 'Daddy' or 'Brother so-and-so, I don't like it when you touch my bum. I don't like it when you touch my breast.' You don't think that is important? I am also an adolescent, that's why I'm talking to you that way.

[Respondent]

I think we can discuss that too.

Because I know there are a lot of people who go out, a man starts touching certain places they don't like, but they can't say it. Before they know it, the man has already slept with them against their will.

[Respondent]

Yes.

And a lot of girls are going through this. They go out, enter into a man's room, enter into a man's shop, and before they know it, the man has already slept with them without their consent because they were shy. It does happen, isn't it?

[Interviewer]

Exactly the point I'm trying to make. So I'm putting it in that you said that when we meet, we should have discussions about communicating your feelings, communicating your hurts, communicating exactly how you feel in your heart, communicating exactly what is going on in your mind. Are you getting what I'm saying?

[Respondent]

Yes.

[Interviewer]

What about adherence? How you can take your drugs promptly?

[Respondent]

Yes, oh, I don't like them.

[Interviewer]

You don't like the drugs? Aha, this is the more reason why we have to talk about how you can take your drugs promptly without missing your drugs. You understand?

[Respondent]

Yes.

I think we should discuss the issues with the drugs and the effects of the drugs.

[Interviewer]

Thank you so much, my dear.

**[Interviewer]**

What other recreational activities, physical activities, or mental activities can we include in the program?

[Respondent]

Hmmm, drumming and playing ampe

[Interviewer]

So can we include any physical activity, recreational activity, or mental activities during the training? Tell me some of the activities that you think we can include in the training that will make the training very interesting for you.

[Respondent]

Like singing, dancing, and playing football.

[Interviewer]

What about ludo or oware?

[Respondent]

Yes, and ampe.

[Interviewer]

What about physical exercise? Maybe you can stand and then you be turning around?

[Respondent]

Yes. That will be very helpful.

[Interviewer]

Great. I have noted all these things you have said, and we are going to make sure we implement them. What support can we give you so that you'll be able to come regularly? Is there anything that you anticipate that can stop you from attending the program regularly? Is there anything that you anticipate—'this thing will not allow me to come for the program'? I want to know so that we can deal with that problem even now.

[Respondent]

Yes, my brothers.

[Interviewer]

Why are you saying your brothers? Why do you think that your brothers can stop you from coming?

[Respondent]

Sometimes if I say they should come and escort me to this hospital, they'll say they are tired.

[Interviewer]

Oh, so sometimes when you want them to escort you to the hospital, they'll tell you they are tired?

[Respondent]

Yes.

[Interviewer]

Okay. So what can we do? What can we do so that we can always get one of your brothers to come with you to the program? What do you think we can do to make sure that every Sunday when we have the program, one of your brothers will come with you?

[Respondent]

You should call him, else he will not come.

[Interviewer]

Okay, so I will get the number of one of your brothers from Auntie Helen or Ma Helen. So that when the program is due, before maybe on Friday or Saturday, we'll be calling your brother to remind him so that he will come with you. But when you also go, tell him about the discussions we have had so that he will be aware that maybe in June we are going to have such a program. Apart from the difficulty that you envisage with your brothers, what other difficulties or challenges will prevent you and your brothers from coming every Sunday for the program?

[Respondent]

That's all the challenge.

[Interviewer]

Which means that I have your assurance that once the program starts every Sunday, you'll be coming with your brothers?

[Respondent]

Yes.

**[Interviewer]**

Thank you so much, my dear. Is there any other thing on your mind that you would want to share with me? Any other thing on your mind? I have told you, you must learn how to communicate exactly how you feel. You must learn how to communicate what you think without fear. Are you getting what I'm saying?

[Respondent]

Yes.

[Interviewer]

So tell me anything on your mind. Do not fear.

[Respondent]

I just want to ask for help with the PTA money that they said we should pay.

[Interviewer]

How much is the PTA?

[Respondent]

70 cedis.

[Interviewer]

70 Ghana cedis?

[Respondent]

Yes. My brothers said they don't have money.

[Interviewer]

Don't worry. I am glad that you have been bold to communicate exactly your needs. Part of the things we will discuss is to learn to communicate your needs to whoever is in charge of taking care of you. If you don't learn how to communicate your needs to your parents, your guardian, your siblings, you will be tempted to go out there to tell one stupid boy or one stupid man who will give you that money in exchange for sex. Are you getting what I'm saying?

[Respondent]

Yes.

[Interviewer]

So I will send the 70 cedis to Ma Helen, and Ma Helen will give it to you the day you come for clinic or today. I will send it to her today. Okay?

[Respondent]

Okay.Thank you so much

[Interviewer]

So that Ma Helen will give you the 70 cedis to go and pay the PTA fee. Alright. So our interview has come to an end. Thank you so much for making time to talk to me. Okay?

[Respondent]

Okay.

[Interviewer]

Alright, bye.

END OF TRANSCRIPT
