# Supplementary material for: Potential enablers for the implementation of multiple family group therapy intervention in the lower Manya Krobo District, Ghana: Perspectives of multiple stakeholders
Source: PLOS Glob Public Health. 2026 Jan 16;6(1):e0005620. doi: 10.1371/journal.pgph.0005620 (PMC12810799; doi:10.1371/journal.pgph.0005620)
Supplement: Attachment — Submitted filename: Response to Editor and Reviewers comment.docx [file pgph.0005620.s008.docx]

Dear Editor,

On behalf of my co-authors, I would like to sincerely thank you for your efforts in handling our manuscript: Potential Enablers for the implementation of multiple family group therapy intervention in the Lower Manya Krobo District, Ghana: Perspectives of multiple stakeholders**.**

. We greatly appreciate the time and attention you devoted to coordinating the peer review process and securing qualified reviewers.

We are particularly grateful for the thoughtful and constructive comments provided by the reviewers. Their insights have been invaluable in refining the methodological rigor, clarity, and overall reporting quality of our manuscript. We believe that the revisions guided by these comments have significantly strengthened the paper, enhancing its contribution to the field.

We appreciate your continued support and guidance throughout this process and look forward to the next steps in the review.

Thank you once again for your dedication and professional oversight.

| **Editors Comments** | **Authors’ Response** | **Page** |
| --- | --- | --- |
| Please amend your online detailed Financial Disclosure statement. This is published with the article. It must therefore be completed in full sentences and contain the exact wording you wish to be published.  a) State the initials, alongside each funding source, of each author to receive each grant, if applicable. For example: "This work was supported by the National Institutes of Health (####### to AM; ###### to CJ) and the National Science Foundation (###### to AM)."  b) State what role the funders took in the study. If the funders had no role in your study, please state: “The funders had no role in study design, data collection and analysis, decision to publish, or preparation of the manuscript.”  For more information, please go to our submission guidelines: <https://journals.plos.org/globalpublichealth/s/submission-guidelines#loc-financial-disclosure-statement> | We thank the editor for this guidance. Our **amended Financial Disclosure statement** is as follows:  **“**This work was supported by [Science for Africa Foundation, POS24-07 to SA]. The funders had no role in study design, data collection and analysis, decision to publish, or preparation of the manuscript.” |  |
| Please ensure that the funders and grant numbers match between the Financial Disclosure field and the Funding Information tab in your submission form. Note that the funders must be provided in the same order in both places as well. | Thank you for your guidance. Please we have carefully reviewed the submission and confirm that the **funders and grant numbers now match exactly** between the Financial Disclosure statement and the Funding Information tab in the submission form. The order of funders has also been aligned in both locations to ensure consistency, as requested. |  |
| Please send a completed ‘Competing Interests’ statement, including any COIs declared by your co-authors. If you have no competing interests to declare, please state “The authors have declared that no competing interests exist”. Otherwise please declare all competing interests beginning with the statement ‘I have read the journal's policy and the authors of this manuscript have the following competing interests:’  For more information, please go to our submission guidelines: <https://journals.plos.org/globalpublichealth/s/submission-guidelines#loc-competing-interests> | Thank you for the guidance. Please we have completed the Competing Interests statement as requested. It reads as follows:  **“The authors have declared that no competing interests exist.”**  This statement confirms that all authors have reviewed the journal’s policy and have no financial or non-financial conflicts to disclose in relation to this manuscript. |  |
| In the online submission form, you indicated that “Information regarding this paper is available upon reasonable request from the corresponding author S.A.”.  All PLOS journals now require all data underlying the findings described in their manuscript to be freely available to other researchers, either  a) In a public repository,  b) Within the manuscript itself, or  c) Uploaded as supplementary information.  This policy applies to all data except where public deposition would breach compliance with the protocol approved by your research ethics board. If your data cannot be made publicly available for ethical or legal reasons (e.g., public availability would compromise patient privacy), please explain your reasons by return email and your exemption request will be escalated to the editor for approval. Your exemption request will be handled independently and will not hold up the peer review process, but will need to be resolved should your manuscript be accepted for publication. One of the Editorial team will then be in touch if there are any issues. | We respectfully request an exemption from public deposition of the underlying data due to the sensitive nature of the study population (adolescents living with HIV) and the ethical considerations surrounding confidentiality and stigma. Making the full dataset publicly available could compromise participant privacy despite de-identification efforts.  In line with PLOS policies, we are committed to transparency and will provide **anonymized data upon reasonable request**. Researchers seeking access to the data may contact the corresponding author directly, who will evaluate requests to ensure compliance with ethical and privacy protections. |  |
| Please ensure that you refer to Table 1 in your text as, if accepted, production will need this reference to link the reader to the table. | Thank you for the feedback. Please we have updated the manuscript to ensure that Table 1 and all other tables are explicitly cited in the main text wherever the sociodemographic characteristics of participants and themes are discussed. | 12-16 |
| We have noticed that you have uploaded Supporting Information files, but you have not included a list of legends. Please add a full list of legends for your Supporting Information files before or after the references list. | Thank you for the guidance. Please we have added a complete list of legends for all Supporting Information files, placed after the References section, to ensure clarity and proper identification of each supplemental file. |  |
| If the reviewer comments include a recommendation to cite specific previously published works, please review and evaluate these publications to determine whether they are relevant and should be cited. There is no requirement to cite these works unless the editor has indicated otherwise. | Thank you for the guidance. We have reviewed the publications suggested by the reviewers and assessed their relevance to our study. While we found some alignment with our work, we have included only those references that substantively support or contextualize our findings, in accordance with the editor’s guidance |  |

| **Reviewer Comments # 1** | **Authors’ response** | **Page** |
| --- | --- | --- |
| This is a culturally appropriate and methodologically robust qualitative study that identifies enabling contexts for MFGT implementation among adolescents living with HIV (ALHIV) in a resource-limited environment. The study is rich in perspectives from participants, primarily through stakeholder triangulation involving caregivers, ALHIV, and health workers. The study presents insightful additions to implementation science in global mental health and HIV treatment, particularly in sub-Saharan Africa. | We sincerely thank the reviewer for the positive and encouraging feedback. We are grateful for your recognition of the methodological rigor, cultural appropriateness, and richness of participant perspectives in our study. Your comments affirm the contribution of our work to implementation science in global mental health and HIV treatment |  |
| 2. Major Strengths Relevance & Originality: Emphasizes a previously unexplored yet critical area, such as mental health care provision for ALHIV in SSA, through a family-based model. Methodologically Rigorous Qualitative Design: Thematic analysis, use of triangulation, and reflexivity are outstanding. Stakeholder Involvement: Incorporation of several perspectives (youth, care providers, caregivers) demonstrates ethical, equitable research Policy Value: Provides in-depth, practical information on program feasibility, adaptation, and cultural considerations. | We sincerely thank the reviewer for highlighting the major strengths of our study. We greatly appreciate your recognition of the relevance and originality of our research, the methodological rigor of our qualitative design, the ethical and equitable inclusion of multiple stakeholder perspectives, and the potential policy value of our findings |  |
| Data Availability and Transparency Issue: The Data Availability Statement is limiting data to "reasonable request," which is not in alignment with PLOS's open data policy. Recommendation: Authors should specify what data (e.g., anonymised transcripts, coding schemes) are available to be shared and post them to a public repository or offer a justified exemption (e.g., participant privacy issues). | We thank the reviewer for the guidance on data availability. Given the sensitive nature of participants’ condition, the stigma associated with HIV, and ethical considerations, we are unable to deposit transcripts in a public repository or share them online. However, in accordance with ethical constraints and to maintain participant confidentiality, anonymized transcripts can be made available upon reasonable request. Individuals seeking access to these materials are invited to contact the corresponding author directly. |  |
| . Analytical Depth and Triangulation Issue: Although the methodology mentions triangulation, a more explicit comparative synthesis is needed across stakeholder groups in the findings. Recommendation: Include a paragraph in the discussion findings mentioning points of consensus or divergence among all three stakeholder groups. | We thank the reviewer for this insightful suggestion. In response, we have added a paragraph in the *Discussion* section explicitly highlighting points of consensus and divergence across the three stakeholder groups—adolescents living with HIV, caregivers, and health workers. This addition emphasizes the comparative synthesis of perspectives, strengthening the analytical depth and the triangulation of our qualitative findings. | 27 |
| Limitations and Reflexivity Issue: The limitations discussion could provide a more precise explanation of how the researcher's positionality may have influenced data interpretation, particularly in light of power differentials. Recommendation: Discuss further reflexivity to explain how training of the research team or institutional status may have affected interaction with vulnerable respondents. | We thank the reviewer for this important suggestion. In response, we have expanded the *Limitations* section to provide a more detailed discussion of reflexivity, explicitly addressing how the researchers’ positionality, institutional affiliation, and prior training may have influenced interactions with participants and the interpretation of data |  |
| D. Conceptual Framing Issue: The CFIR is mentioned in tool development, but not meaningfully integrated into the analysis or discussion.  Recommendation: Briefly map the five CFIR domains (intervention characteristics, outer setting, inner setting, individuals, and implementation process) to the identified themes to enhance theoretical clarity. | We thank the reviewer for this insightful suggestion. In response, we have incorporated a brief mapping of the five CFIR domains—**intervention characteristics, outer setting, inner setting, individuals, and implementation process**—to the identified themes in the *Results*. | 12-26 |
| Describe what is meant by "Form 4" in Table 1, caregiver education. | We thank the reviewer for this observation. We have clarified in Table 1 that *“Form 4”* is equivalent to the former Ordinary-level in the previous Ghanaian education system. |  |
| Some sentences are too long and could be condensed; a slight edit to improve clarity and flow is preferable. Ensure consistency in referencing styles; some in-text citations use the author-date format, while others use the numeric format | We thank the reviewer for this helpful feedback. We have carefully revised the manuscript to condense overly long sentences, improving clarity and readability. Additionally, we have ensured consistency in referencing style throughout the manuscript, standardizing all in-text citations to the numeric format in accordance with the journal’s guidelines. |  |
|  | Authors’ response | Page |
| Reviewer # 2 |  |  |
| "There are notable enablers of the MFGT for ALHIV in Lower Manya Krobo District. Researchers and practitioners are advised to pay critical attention to enablers in their quest to implement MFGT to address the mental health burden of ALHIV". This conclusion does not summary the findings, Researchers and practitioners are advised to pay................ is not an actionable recommendation. Kindly provide an actionable recommendation | We thank the reviewer for this constructive feedback. In response, we have revised the conclusion to more clearly summarize the key findings and provide actionable recommendations. The revised text highlights specific enablers identified in the study—such as stakeholder consensus on privacy, culturally adapted scheduling, and integration of spiritual elements—and recommends that implementers design flexible MFGT programs that incorporate these factors while addressing divergences in stakeholder preferences to enhance engagement and effectiveness. | 2 |
| Keywords: Multiple Family Group Therapy; implementation science; adolescents living with HIV; perspectives; multiple stakeholders these are not keyword, they are too long | We thank the reviewer for this helpful comment. We would like to clarify that Medical Subject Headings (MeSH) allow the use of 2–3 word phrases to represent a single concept for the purposes of indexing, cataloging, and searching biomedical and health-related information. Accordingly, our revised keywords combine relevant phrases to ensure accurate indexing while remaining concise and aligned with MeSH conventions. | 2 |
| This study lacks Theoretical issues | We respectfully disagree with the characterization that the study lacks theoretical grounding. The study is explicitly informed by the **Consolidated Framework for Implementation Research (CFIR)**, which guided both the development of data collection tools and the interpretation of findings. CFIR provides a well-established implementation science framework that addresses intervention characteristics, inner and outer settings, individual characteristics, and implementation processes. We have however included a subsection “ Theoretical grounding of the study” in the main text. | 5 |
| Study population cannot be seen in the study and exclusion is also missing | We respectfully note that details on the study population, inclusion, and exclusion criteria are already provided in the main text under the subheading *“Research Participants and Sampling.”* This section clearly describes the participants, their characteristics, and the criteria used for inclusion and exclusion in the study. Please see page 6. | 6 |
| Sample sizes were determined based on achieving theoretical saturation while remaining logistically feasible. The final sample included 12 ALHIV, 13 caregivers, and 5 healthcare professionals, for a total of 30 participants. This multi-stakeholder approach allowed for triangulation of perspectives and comprehensive understanding of implementation enablers from different vantage points within the healthcare ecosystem.......... This is not well justified | We thank the reviewer for this observation. We have revised the *Methods* section to provide a more detailed justification for the sample size. Specifically, we explain that the number of participants was guided by the principle of **theoretical saturation**, ensuring that data collection continued until no new themes emerged across stakeholder groups. Additionally, the sample size balanced **practical and logistical considerations** while maintaining sufficient diversity across adolescents living with HIV, caregivers, and healthcare professionals to enable robust triangulation of perspectives and a comprehensive understanding of implementation enablers within the healthcare ecosystem. | 7 |
| Research team is also missing, who were the research team. Do all team members had prior experience with qualitative interviews?. training session ensured consistency in the use of research instruments and interview techniques is also missing in the work. The recruitment period for this study started is also missing in the work | We thank the reviewer for this observation. We have updated the *Methods* section to provide detailed information on the research team, including their roles and prior experience with qualitative interviews. Additionally, we have described the training sessions conducted to ensure consistency in the use of research instruments and interview techniques across all team members. Finally, we have included the start and end dates of the recruitment period to provide a complete timeline for the study. | 7-9 |
| Data collection occurred through two distinct modalities. 6. "Individual interviews were conducted in English by the 1st author using remote methods, either via telephone or Google Meet videoconferencing platform" what if there was network issues how did you address such and why online interview do you have any justification for it?. | We thank the reviewer for this important query. The decision to conduct individual interviews online (via telephone or Google Meet) was guided by practical considerations, including participants’ privacy, convenience, and minimizing disruptions to their daily schedules. To address potential network issues, backup strategies were implemented: interviews were rescheduled if connectivity problems occurred, and participants were offered telephone calls as an alternative to videoconferencing when video quality was poor. This flexible approach ensured that data collection could proceed without compromising the quality of participant engagement or the completeness of responses, while maintaining confidentiality and comfort for adolescents living with HIV. | 8 |
| FGDs lasted approximately 90-120 minutes, while interviews lasted 60-90 minutes. You mean the interview last more than an hour? | We thank the reviewer for this observation. Yes, individual interviews typically lasted **60–90 minutes**, depending on participant responses and depth of discussion. We have clarified this in the *Methods* section to ensure readers understand that interviews were conducted in a flexible manner to allow participants sufficient time to share their perspectives comprehensively. | 8 |
| Challenges encountered. What was the challenges encounter in this study | We thank the reviewer for this comment. We have added a section in the *Methods* describing the challenges encountered during the study, including logistical issues such as scheduling conflicts, occasional network interruptions during online interviews, language translation complexities, and managing participant comfort and confidentiality. We also describe the strategies implemented to mitigate these challenges, ensuring the quality and integrity of data collection. | 8-9 |
| Trustworthiness and rigor. You cannot write a full write on Trustworthiness and rigor without citing a study or without using the Consolidated Criteria for Reporting Qualitative Research (COREQ) | We thank the reviewer for this observation. We have revised the *Trustworthiness and Rigor* section to explicitly reference relevant literature and aligned our reporting with the **Consolidated Criteria for Reporting Qualitative Research (COREQ)** checklist. | 10-11 |
| Table 1: Sociodemographic characteristics of participants. Table 1 is AI generated please can justify why you are using AI generated table. The table is even scattered. the table should be simple. eg. Variables - Frequency - Percentage rather than the AI generated table | We thank the reviewer for this observation. We acknowledge that the original table was generated using AI for initial formatting purposes, which resulted in a scattered presentation. We have revised Table 1 to a simpler and clearer format, presenting participant sociodemographic characteristics with the columns **“Variable,” “Frequency,” and “Percentage** |  |
| Thematic Result The presentation of the result is not appropriate. Where is your thematic table. The thematic table should consist of the main theme, sub theme and codes before interpretation of the results with each sub theme. How many sub theme and codes were generated ?. All these needs to appear in the work. Kindly see this qualitative work from plos mental health to restructure your result <https://journals.plos.org/mentalhealth/article?id=10.1371/journal.pmen.0000310> and you can also use this link as well <https://pmc.ncbi.nlm.nih.gov/articles/PMC12224659/> 3. Re structure all the result section again | We thank the reviewer for this constructive feedback. In response, we have restructured the Results section to include a **comprehensive thematic table** prior to interpretation. The table now clearly presents:**Main Themes, Sub-Themes, Codes and has been organized across the five CFIR domains** | 15-16 |
| The discussion is not good. You need write a summary of the results then u compare it to other studies then you tell us what implies and what your study suggest. it is not about discussing other literatures. Please kindly re-write your discussion again | We thank the reviewer for this insightful feedback. We have thoroughly revised the *Discussion* section to improve its structure and clarity. The revised discussion now:   1. **Summarizes the key study findings** upfront, highlighting main themes, sub-themes, and stakeholder perspectives. 2. **Compares these findings with relevant existing literature**, emphasizing similarities and differences, while situating our results within the broader context of MFGT and mental health interventions for ALHIV. 3. **Explores the implications of the findings**, detailing what they suggest for practice, policy, and future research. 4. **Clearly identifies actionable recommendations** for implementation strategies and cultural adaptation, rather than solely reviewing prior studies. | 29-32 |
|  |  |  |
| Reviewer #3 |  |  |
| Your paper demonstrates sound methodological and ethical rigor, presents conclusions appropriately supported by the data, and makes a significant contribution to global public health by addressing the implementation of a psychosocial intervention for adolescents living with HIV (ALHIV) in a resource-limited setting. | We sincerely thank the reviewer for this encouraging feedback. We appreciate the recognition of the methodological and ethical rigor of our study, as well as the relevance of our findings to global public health. We are glad that the study’s contribution to understanding the implementation of psychosocial interventions for adolescents living with HIV in resource-limited settings is acknowledged and valued. |  |
| The recommendations for implementation (hybrid model, diverse facilitators, cultural adaptations) are comprehensive but presented without clear prioritization. To enhance practical utility, you can consider briefly indicating which recommendations are most critical or feasible based on the data. | We thank the reviewer for this valuable suggestion. In response, we have revised the recommendations section to indicate **priority and feasibility** of the suggested implementation strategies. Specifically, we highlight that culturally adapted interventions and strategies ensuring privacy and confidentiality are most critical, while approaches such as the hybrid model and diverse facilitator involvement are feasible enhancements that can be implemented progressively | 33 |
| Also expand the discussion to explicitly address how findings could inform implementation in similar SSA settings. | Thank you for this feedback. The discussion section has been revised to explicitely address how findings could inform implementation in similar SSA settings | 29-32 |
|  |  |  |
| Reviewer # 4 |  |  |
| The author did quite well in identifying the enablers for MFGT implementation in the Lower Manya Krobo District, Ghana. The following points are my suggestions regarding this work: | We sincerely thank the reviewer for acknowledging the strengths of our study and for recognizing our efforts in identifying enablers for MFGT implementation in the Lower Manya Krobo District. We appreciate the thoughtful suggestions provided and have carefully considered them in revising the manuscript to enhance clarity, rigor, and practical relevance. |  |
| In the first paragraph of the introduction, the statement: Previous studies has... should read: Previous studies have... | We thank the reviewer for this correction. The sentence has been revised to read: **“Previous studies have…”** to ensure proper subject-verb agreement. | 2 |
| Table 1 is too long; it is suggested to be divided into three parts. Remove the column with the heading 'Population Group', which contains repeated names of subpopulation groups. Each table should have a heading, for example: Table 1: Sociodemographic Characteristics of Adolescents Living with HIV Table 2: Sociodemographic Characteristics of Healthcare Workers Table 3: Sociodemographic Characteristics of Caregivers | We thank the reviewer for this suggestion. In response, we have **divided the original Table 1 into three separate tables**, each focusing on a distinct participant group. The column “Population Group” has been removed to reduce redundancy. The revised tables are now labeled as follows:   - **Table 1:** Sociodemographic Characteristics of Adolescents Living with HIV - **Table 2:** Sociodemographic Characteristics of Healthcare Workers - **Table 3:** Sociodemographic Characteristics of Caregivers |  |
| On page 21, the participant ALHIV 1 articulated the statement: "So, you will have to talk more about the suicidal thoughts we have." In the explanation that follows this statement, the author should add the need to address suicidal thoughts being experienced by the ALHIV | We thank the reviewer for this important observation. We have revised the text following the participant quote to explicitly highlight the need to **address suicidal thoughts experienced by ALHIV** | 27 |
| Page 22: The statement, "I think we role-playing, how we can give support to our parents and siblings, and how we can receive support would help us," is not properly phrased, especially the first part of the statement. The author should review the way the statement is presented for more clarity. | We thank the reviewer for highlighting this phrasing issue. The statement has been revised for clarity to read:"*I think role-playing how we can give support to our parents and siblings and also how we can receive support would help us"* | 28 |
