# Supplementary material for: Potential enablers for the implementation of multiple family group therapy intervention in the lower Manya Krobo District, Ghana: Perspectives of multiple stakeholders
Source: PLOS Glob Public Health. 2026 Jan 16;6(1):e0005620. doi: 10.1371/journal.pgph.0005620 (PMC12810799; doi:10.1371/journal.pgph.0005620)
Supplement: Attachment — Submitted filename: Response to Reviewers comment_PGPH.docx [file pgph.0005620.s009.docx]

| **Reviewer’s comment** | **Authors response** | **Page** |
| --- | --- | --- |
| This revised paper is a fascinating contribution to the literature on family-centered mental health interventions among adolescents living with HIV (ALHIV) from sub-Saharan Africa. That you managed to integrate the perspectives of numerous stakeholders and culture-bound strategies for determining feasibility earns accolades | Thank you for the compliments |  |
| Triangulation and Comparative Synthesis: You increased the integration of viewpoints within the different groups of stakeholders. Additional synthesis within the text clarifies the argument. A brief comparative table summarizing the extent of consensus and disagreement among caregivers, ALHIV, and health workers would facilitate the reader's use. | We sincerely appreciate the reviewer’s insightful suggestion. In response, we have developed a comparative summary table (see **Table 5**) that highlights the areas of convergence and divergence in perspectives among caregivers, adolescents living with HIV (ALHIV), and health workers. This table complements the narrative synthesis by providing a concise visual overview of consensus and disagreement across key themes, thereby enhancing the clarity and accessibility of the findings. Corresponding explanations have also been added to the results section | 17-18 |
| Reflexivity and Positionality: Reflexivity was slightly widened. It would be desirable, though, to describe briefly the degree to which the positionality of the researchers (e.g., professional experience, institutional affiliation, cultural familiarity with participants’ culture) affected interaction with the participants or the interpretation of the answers—particularly considering the power imbalance involved when working with vulnerable groups of adolescents. | We thank the reviewer for this valuable comment. In response, we have expanded the section on reflexivity to include a more explicit discussion of the researchers’ positionality | 10-11 |
| CFIR Integration: The reference to CFIR on your tool development is helpful. To enhance theoretical clarity, we would like you to succinctly map your results to the five CFIR domains in a summary paragraph or a very short table within the Discussion section. Doing so would make the study more relevant for implementation science experts. | We appreciate the reviewer’s thoughtful comment. However, we had already provided a detailed thematic mapping of our findings to the five CFIR domains—**intervention characteristics, outer setting, inner setting, characteristics of individuals, and process**—to strengthen theoretical clarity and relevance to implementation science. This mapping was presented in **Table 4 (pages 15–17)**, which succinctly summarizes how each major theme aligns with the respective CFIR domains. A brief explanatory paragraph has also been added to the Discussion section to guide readers’ interpretation. | 15-17, 20-31, 32 |
| Ethical Considerations and Data Transparency: Include a statement ensuring IRB/ethics approval was granted or waived, and by what institution. Discuss the way participant consent and confidentiality were managed, particularly for adolescents. | We appreciate the reviewer’s attention to ethical and data transparency issues. We respectfully note that the requested information is already included in the manuscript. Specifically, the **Ethical Considerations** section (pages 11-12) states that the study received ethical approval from the Ghana Health Service Ethics Review Committee (GHS-ERC:004/07/24) | 11-12 |
| The current Data Availability Statement doesn't quite align with PLOS policy. If you're unable to make all your data publicly available, please explain (e.g., due to the terms of ethical approval or participant anonymity). Alternatively, deposit a de-identified codebook or summary data into a repository. | We thank the reviewer for this important observation. In accordance with the ethical requirements set forth by our Institutional Review Board (**[insert name of ethics committee/institution, e.g., Institutional Review Board of the University of Education, Winneba (Approval No. XXX)]**), we are not permitted to make participants’ full transcripts publicly available or deposit them in a data repository, as this could compromise confidentiality and violate the conditions of ethical approval—particularly given the sensitivity of the study population (adolescents living with HIV).  However, to support transparency and reproducibility, **de-identified summary data and codebook** describing the main themes and variables can be made available **upon reasonable request** to the corresponding author. The **Data Availability Statement** has been revised accordingly to reflect this clarification. | 39 |
| Ensure consistency of referencing style (author-date or numerical). | We thank the reviewer for this helpful observation. We have thoroughly reviewed the entire manuscript to ensure consistency in referencing style. All in-text citations and reference list entries have now been formatted according to the **[insert required style, e.g., PLOS numerical referencing style]**, ensuring uniformity throughout the manuscript. |  |
| Define such items as the table entry for "Form 4" for foreign readers, which you've just done, | We thank the reviewer for this observation. However, “Form 4” has already been defined in the manuscript to read “ordinary level” as recommended by the previous reviewers to aid understanding among international readers. | 14 |
| concise sentences where possible for greater clarity. | We appreciate the reviewer’s constructive feedback. In response, we have carefully revised the manuscript to improve readability by shortening and restructuring lengthy sentences. Redundant phrasing has been removed, and key ideas are now expressed more concisely to enhance clarity and flow throughout the text. |  |
| Check the usage of acronyms and spell them out on first occurrence. | We thank the reviewer for this helpful suggestion. We have carefully reviewed the entire manuscript to ensure that all acronyms are **spelled out in full at their first occurrence**, followed by the abbreviation in parentheses. Subsequent mentions now consistently use only the acronym. |  |
